# Laser Fabrication of Multi‐Dimensional Perovskite Patterns with Intelligent Anti‐Counterfeiting Applications

**DOI:** 10.1002/advs.202309862

**Published:** 2024-08-09

**Authors:** Xiangyu Xu, Shoufang Liu, Yan Kuai, Yuxuan Jiang, Zhijia Hu, Benli Yu, Siqi Li

**Affiliations:** ^1^ School of Physics and Optoelectronic Engineering Anhui University Hefei Anhui 230601 P. R. China; ^2^ Information Materials and Intelligent Sensing Laboratory of Anhui Province Anhui University Hefei Anhui 230601 P. R. China; ^3^ Key Laboratory of Opto‐Electronic Information Acquisition and Manipulation of Ministry of Education Anhui University Hefei Anhui 230601 P. R. China

**Keywords:** anti‐counterfeiting, artificial intelligence, femtosecond laser direct writing, perovskite

## Abstract

Perovskites have gained widespread attention across various fields such as photovoltaics, displays, and imaging. Despite their promising applications, achieving precise and high‐quality patterning of perovskite films remains a challenge. In this study, femtosecond laser direct writing technology is utilized to achieve rapid and highly precise micro/nanofabrication on perovskites. The study successfully fabricates multiple structured and emission‐tunable perovskite patterns composed of A_2_(FA)_n−1_Pb_n_X_3n+1_ (A represents a series of long‐chain amine cations, and X = Cl, Br, I), encompassing 2D, quasi‐2D, and 3D structures. The study delves into the intricate interplay between fabrication technology and the growth of multi‐dimensional perovskites: higher repetition rates, coupled with appropriate laser power, prove more conducive to perovskite growth. By employing precise halogen element design, the simultaneous generation of two distinct color quick‐response (QR) code patterns is achieved through one‐step laser processing. These mirrored QR codes offer a novel approach to anti‐counterfeiting. To further enhance anti‐counterfeiting capabilities, artificial intelligence (AI)‐based methods are introduced for recognizing patterned perovskite anti‐counterfeiting labels. The combination of deep learning algorithms and a non‐deterministic manufacturing process provides a convenient means of identification and creates unclonable features. This integration of materials science, laser fabrication, and AI offers innovative solutions for the future of security features.

## Graphical abstract

Femtosecond laser direct writing (FsLDW) is employed to fabricate emission‐tunable perovskite patterns with 2D, quasi‐2D RP, and 3D structures. The technique ensures precise and rapid micro/nanofabrication, creating dual‐color QR code patterns for anti‐counterfeiting. By integrating artificial intelligence for pattern recognition, the study significantly enhances security features, providing an advanced, unclonable anti‐counterfeiting solution for future applications.

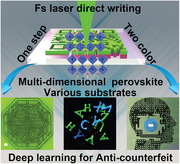

## 1 Introduction

Metal halide perovskites are regarded as future “stars” materials in various applications due to their exceptional optoelectronic properties, such as high light absorption coefficients and superior charge carrier mobility.^[^ ^1^, ^2^, ^3^, ^4^ ^]^ These materials are particularly competitive in display and imaging technologies, showcasing exceptional potential for the development of high‐resolution and highly sensitive displays.^[^ ^5^, ^6^, ^7^, ^8^ ^]^ This inherent capability renders them attractive for applications in anti‐counterfeiting and security.^[^ ^9^, ^10^ ^]^ For instance, Tian et al. demonstrate the use of perovskite microdots for encryption.^[^ ^11^ ^]^ Sun et al. highlight the potential of perovskite‐based patterns for secure labeling.^[^ ^12^, ^13^ ^]^ Despite their outstanding performance, considerable challenges remain in achieving high‐precision, high‐quality, and rapid fabrication of perovskite patterned films.^[^ ^14^, ^15^, ^16^, ^17^, ^18^ ^]^

In recent years, femtosecond (fs) laser processing technology has played a vital role in the processing and manufacturing of perovskite materials.^[^ ^19^, ^20^ ^]^ The extremely short pulse duration and ultra‐high peak intensity of fs lasers effectively minimize the formation of heat‐affected zones.^[^ ^21^, ^22^, ^23^ ^]^ This characteristic provides significant advantages for perovskite fabrication, including high precision, remarkable flexibility, and efficiency, which enables rapid and large‐area patterning.^[^ ^24^, ^25^, ^26^, ^27^ ^]^ The interaction mechanisms between lasers and perovskites are primarily categorized into laser‐induced crystallization and laser ablation. Due to their low formation energies and ionic properties, 3D perovskites can be readily crystallized through the thermal effects of laser irradiation. Notably, Huang et al. demonstrated the in situ reversible growth and erasure of CsPbBr_3_ quantum dots within a glass matrix using an fs laser with varying power densities.^[^ ^28^ ^]^ Liang et al. achieved high‐resolution in situ crystallization and patterning of CsPbBr_3_ films for light‐emitting diodes application through fs laser printing.^[^ ^25^ ^]^

For low‐dimensional perovskite structures, such as 2D perovskites, a common approach involves pre‐crystallizing the perovskite films followed by high‐power laser ablation to create specific patterns.^[^ ^29^ ^]^ Initially, pulsed energy is rapidly deposited, generating electrons through photon ionization. These electrons are then heated and transferred to the perovskite lattice, melting the solid perovskite into a superheated liquid via thermalization. When the ablation threshold is surpassed, the vaporization of the perovskite material results in the formation of a hole at the laser‐focused spot, effectively removing the material.^[^ ^30^, ^31^ ^]^ Despite the success of these techniques, there remains a notable gap in the use of fs laser methods for directly inducing low‐dimensional perovskite crystallization.

In this study, our objective is to employ fs laser direct writing (FsLDW) technology for the precise patterning of perovskites across various dimensions and to investigate the nucleation and growth mechanism induced by perovskite‐light interaction. To accomplish this, we have developed a novel perovskite polymer hybrid system composed of a series of A_2_(FA)_n−1_Pb_n_X_3n+1_, where “n” ranges from 1 to ∞. Here, “A” represents long‐chain amine cations such as 5‐aminovaleric acid hydrobromide (AVA^+^), phenethylamine (PEA^+^), 1‐Naphthylmethylamine (NMA^+^), 4‐phenylbutan‐1‐amine hydrobromide (PhBA^+^), 1‐methyl‐4,5‐dihydro‐1h‐imidazol‐2‐amine hydrobromide (MDI^+^), and alpha‐amino‐gamma‐butyrolactone hydrobromide (ABL^+^). “FA” denotes formamidinium ion (HC(NH_2_)^2+^), and “X” represents halide elements such as chlorine (Cl), bromine (Br), and iodine (I). Through specialized preheating treatment, we transformed this hybrid system into transparent perovskite precursor/polyacrylonitrile (PAN) films for subsequent laser processing. Our findings indicate that higher repetition rates, in conjunction with appropriate laser power densities, are more effective for perovskite growth, while excessive energy and heat accumulation can hinder successive growth. Leveraging the self‐supporting nature and thermoplasticity of PAN polymers, we achieved intricate processing of free‐standing 2D, quasi‐2D RP, and 3D perovskite patterns, which were printed on various substrates, including quartz, silicon wafers, and flexible polydimethylsiloxane (PDMS). The polymer encapsulation enhances and ensures the long‐term stability of perovskites.

The versatility and convenience of FsLDW, combined with the enhanced stability of the perovskites, are especially valuable for applications in anti‐counterfeiting. Our technology enables the creation of distinctive perovskite patterns on tickets, and the simultaneous generation of “two distinct color quick‐response (QR) code patterns” through “one‐step laser processing” offers a novel anti‐counterfeiting method. Additionally, we have integrated artificial intelligence (AI)‐based methods for identifying patterned perovskite anti‐counterfeiting labels from micro‐hole array images. This integration of AI technology with materials science and laser fabrication opens new avenues for progress in anti‐counterfeiting technologies.

## 2 Results and Discussion

### 2.1 Fabrication and Processing of Perovskite Precursor Thin Films for Anti‐Counterfeiting Applications

**Figure 1:**
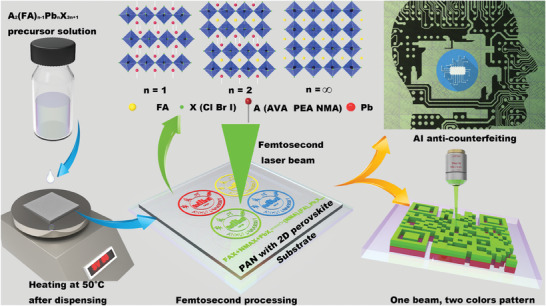
The key procedures conducted in this study involved the following steps. Initially, perovskite precursor solutions were prepared with varying ratios of PAN/DMF solution and deposited onto heated glass substrates to create perovskite precursor films. Subsequently, we utilized a 517 nm fs laser processing system to fabricate 2D, quasi‐2D RP, and 3D perovskite patterns, achieving the production of two‐color perovskite patterns in a single processing step. In addition, we incorporated AI‐based techniques for the utilization of perovskites in anti‐counterfeiting applications.

**Figure 2:**
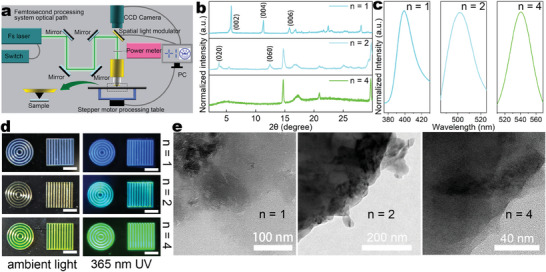
Characterization of 2D, and quasi‐2D RP A_2_(FA)_n−1_Pb_n_Br_3n+1_ perovskite patterns. a) The setup of the fs processing system: the 517 nm fs laser is focused through an objective lens, and processing is achieved by computer‐controlled movement of the stage. b)The XRD patterns of (AVA)_2_(FA)_n−1_Pb_n_Br_3n+1_ films. c) The PL intensities of (AVA)_2_(FA)_n−1_Pb_n_Br_3n+1_ films. d) The (AVA)_2_(FA)_n−1_Pb_n_Br_3n+1_ films with n = 1, n = 2, and n = 4 were fabricated by FsLDW on glass substrates under ambient light and 365 nm UV light, scale bar is 1 mm. e) TEM images of (AVA)_2_(FA)_n−1_Pb_n_Br_3n+1_ films.

The key procedures conducted in this study is shown in **Figure** **1**. Initially, a transparent precursor film was created by drop‐coating the A_2_(FA)_n−1_Pb_n_X_3n+1_/PAN precursor solution, followed by controlled heating at an appropriate temperature. These precursor films, when in a standard environment, cannot spontaneously nucleate perovskite crystals (as shown in Figure S1, Supporting Information). The films were then processed using a 517 nm fs laser (192 fs pulse‐width), with carefully optimized energy density, repetition rate, and speed. **Figure** **2**a illustrates an illustrative representation of a typical FsLDW system used for perovskite growth and patterning. This system comprises a fs laser source, a spatial light modulator system, a microscope objective, and a high‐precision XYZ translation stage, all under computer‐controlled 3D sample stages. This setup enables real‐time monitoring and meticulous control of the fabrication process, facilitating precise processing at micro‐ and nanoscales.

By adjusting the long‐chain amines proportion in the precursor solution, we successfully synthesized diverse perovskite structures, including 2D, quasi‐2D RP, and 3D perovskites. The “n” value was modulated by adjusting the ratios of AX (A = AVA, PEA, NMA, PhBA, MDI, ABL), FAX, and PbX_2_ (X = Cl, Br, I), following the reaction equation:

(1) $$\mathrm{FAX}+2\mathrm{AX}+\mathrm{Pb}X_{2}\to A_{2}{\left(\mathrm{FA}\right)}_{n-1}Pb_{n}X_{3n+1}$$

Maintaining a 2:1 ratio of AX to PbX_2_ while minimizing the FA concentration results in the formation of pure 2D perovskites with an inorganic layer “n” equal to 1. Conversely, adopting a 1:2 AX to FAX ratio leads to an inorganic layer “n” of 2, resulting in the formation of quasi‐2D RP perovskites. Achieving pure 3D perovskites necessitates approaching an AX ratio close to 0.

### 2.2 Characterization of A_2_(FA)_n−1_Pb_n_Br_3n+1_ Thin Film Materials

We first explored the suitability of different long‐chain amines in 2D and quasi‐2D RP, A_2_(FA)_n−1_Pb_n_Br_3n+1_ systems. For preliminary characterization, we performed coarse processing on AVA, PEA, and NMA systems using the fs laser processing system. Figure 2b presents XRD patterns of (AVA)_2_(FA)_n−1_Pb_n_Br_3n+1_ (n = 1, 2, 4) perovskites, revealing diffraction peaks at 5.8°, 11.3°, and 16.8° for n = 1, corresponding to the (002), (004), and (006) planes respectively. For n = 2, the pattern shows peaks at 3.7° and 12.4°, aligned with the (020) and (060) planes. These periodic peaks confirm the regular stacking of octahedra in low‐dimensional perovskites.^[^ ^32^ ^]^ Figure 2c shows the photoluminescence (PL) spectra of (AVA)_2_(FA)_n−1_Pb_n_Br_3n+1_ (n = 1, 2, 4). As “n” increases, the proportion of long‐chain amines decreases, resulting in a shift of the PL peak shift from 399 nm to 541 nm. To further illustrate this point, we fabricated periodic square lines and concentric circles using (AVA)_2_(FA)_n−1_Pb_n_Br_3n+1_ films with different “n” values (n = 1, n = 2, and n = c), as shown in Figure 2d. Additionally, Figure 2e provides a TEM image of the (AVA)_2_(FA)_n−1_Pb_n_Br_3n+1_ film, revealing that the perovskite is well encapsulated by the polymer.

Similarly, we fabricated and characterized (PEA)_2_(FA)_n−1_Pb_n_Br_3n+1_ system. As shown in Figure S3 (Supporting Information), when n = 1, the XRD pattern of (PEA)_2_(FA)_n−1_Pb_n_Br_3n+1_ exhibits diffraction peaks at 5.3°, 10.6°, 15.9°, and 21.3°, corresponding to the (002), (004), (006) and (008) planes, respectively. For n = 2, the diffraction peaks appear at 5.2°, 10.7°, 16.4°, and 21.4°, corresponding to the (002), (004), (006), and (008) planes, respectively. Figure S4 (Supporting Information) shows the PL spectra of (PEA)_2_(FA)_n−1_Pb_n_Br_3n+1_ (n = 1, 2, 4). As “n” increases, the proportion of long‐chain amines decreases, and the PL peak shifts from 412 to 532 nm. The pattern of PEA is shown in Figure S5 (Supporting Information).

We applied the same processing method to other amines, including PhBA, MDI, and ABL to fabricate n = 2 perovskites of the A_2_(FA)_n−1_Pb_n_Br_3n+1_ system. Additionally, we explored mixed systems containing both NMA and PEA amines. Under 365 nm UV light, these films exhibited different shades of blue and green luminescence, highlighting the versatility and adaptability of our system and processing method for various amines (shown in Figure S6, Supporting Information). For the subsequent processing, we selected NMA as the representative long‐chain amine due to its unique luminescent properties. during its luminescence process, when the “n” value is relatively low, the triplet exciton energy level of NMA is lower than that of the [PbBr_6_]^4−^ octahedron in 2D perovskite. The triplet energy transfer process quenches the PL of the perovskite phase, purifying the color of the emission light.^[^ ^10^, ^33^, ^34^ ^]^ This characteristic makes it easier to compare the effects of fs laser processing parameters, such as pulse duration and scanning speed, on the growth of perovskites.

**Figure 3:**
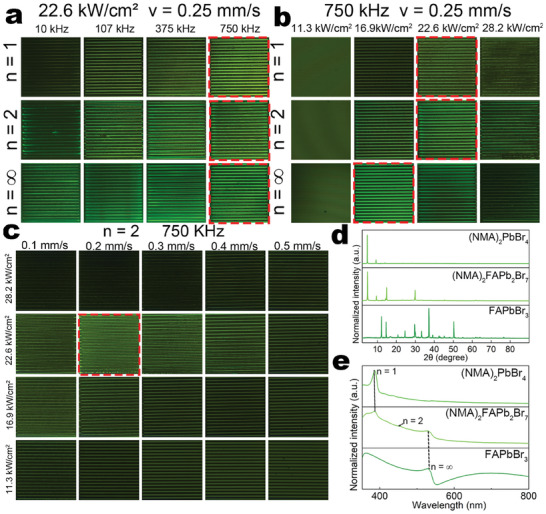
a) 1 × 1 mm squares processed with fs laser at various laser power densities under a repetition rate of 750 kHz. b) 1 ×1 mm squares processed with fs laser at a laser power density of 22.6 kW cm^−2^ and a scanning speed of 0.25 mm s^−1^ under different repetition rates. c) The 1 × 1 mm squares of 2D perovskites were processed with fs laser at different laser powers and scanning speeds under a repetition rate of 750 kHz. d) XRD patterns of (NMA)_2_(FA)_n−1_Pb_n_Br_3n+1_ films. e) UV–vis absorption spectra of (NMA)_2_(FA)_n−1_Pb_n_Br_3n+1_ films.

Before initiating the micropatterning fabrication process, it is crucial to determine the fabrication parameters suitable for different “n” values in the A_2_(FA)_n−1_Pb_n_Br_3n+1_ series. These parameters include the repetition rate, energy power densities, and scanning speed. Initially, the fs laser was set to a repetition rate of 750 kHz and a scanning speed of 0.25 mm s^−1^, with power density variations of 11.3, 16.9, and 22.6 to 28.2 kW cm^−2^ (the numerical aperture of the objective lens was NA = 0.45 and the focal diameter was 15 µm). Employing these parameters, we created a 1 × 1 mm rectangular periodic array and compared their emission to investigate the influence of different power density intensities on the growth of 2D, quasi‐2D RP, and 3D perovskites. As depicted in **Figure** **3**a, at an average power density of 22.6 kW cm^−2^, the brightness of the processed 2D and quasi‐2D RP perovskite films, when illuminated by a UV lamp, was the highest, indicating optimal conditions for perovskite growth. In contrast, pure 3D perovskites exhibited optimal growth at a lower power density of 16.9 kW cm^−2^. The lower energy density for 3D perovskites implies a lower nucleation and growth condition compared to 2D and quasi‐2D RP perovskites. It is noteworthy that excessively high laser power densities (22.6 kW cm^−2^) would damage the generated perovskites due to excessive heat, while excessively low power densities hindered complete perovskite growth. Both of these scenarios are unfavorable for subsequent micro‐nano‐patterning.

We then maintained an average laser power density of 22.6 kW cm^−2^ and a scanning speed of 0.25 mm s^−1^ to explore the influence of different laser repetition rates on perovskite growth (Figure 3b). As the repetition rate decreased, the brightness of the perovskite films under UV light gradually diminished, and more areas of the perovskite were damaged during fs laser scanning. This phenomenon can be attributed to the escalation of pulse energy in the FsLDW process as the repetition rate decreased, leading to excessive energy on the perovskite precursor film over a short period and causing perovskite damage. This result suggests that higher repetition rates with appropriate pulse energy are more favorable for perovskite growth.

Subsequently, we fixed the optimal repetition rate (750 kHz) to explore the effects of scanning speed on perovskite patterning (Figure 3c; Figures S7 and S8, Supporting Information). Various laser power densities (28.2, 22.6, 16.9, and 11.3 kW cm^−2^) were selected, and scanning speeds were set at 0.1, 0.2, 0.3, 0.4, and 0.5 mm s^−1^, aiming to investigate the best scanning speed for perovskites of different dimensions. The results determined the optimal growth parameters for 2D perovskites ((NMA)_2_PbBr_4_) as 22.6 kW cm^−2^ and a scanning speed of 0.1 mm s^−1^. For 2D RP perovskites ((NMA)_2_FAPb_2_Br_7_), the ideal growth parameters were 22.6 kW cm^−2^ and a scanning speed of 0.2 mm s^−1^. The optimal growth parameters for pure 3D perovskites (FAPbBr_3_) were identified as 22.6 kW cm^−2^ and a scanning speed of 0.5 mm s^−1^. We verified the accuracy of our optimal parameters using mathematical methods, as shown in Figure S9 (Supporting Information).

The XRD patterns, illustrated in Figure 3d, showed peaks at 4.6°, and 9.6° indicative of 2D perovskites, and peaks at 14.9° for the n = 2 and n = ∞ samples, associated with 3D perovskites.^[^ ^35^, ^36^, ^37^ ^]^ These findings suggest that a decrease in long‐chain amine content leads to a corresponding reduction in the proportion of 2D perovskites (The small‐angle XRD image is shown in Figure S10, Supporting Information). To gain further insight into the optical properties of the fabricated perovskites, room‐temperature absorption and emission measurements were conducted. The UV–vis absorption spectra revealed distinct characteristic peaks (Figure 3e). Specifically, the spectrum of pure (NMA)_2_PbBr_4_ (n = 1) exhibited a prominent excitonic absorption at 387 nm, while 3D FAPbBr_3_ showed inter‐band absorption near 529 nm. In contrast, the spectrum for n = 2 displayed a strong excitonic absorption at 385 nm, accompanied by additional shoulder peaks at 454 and 529 nm, indicating the coexistence of mixed phases of different dimensions.^[^ ^35^, ^37^ ^]^ Notably, according to the UV–vis absorption spectra (Figure 3e), the fabricated 3D perovskites exhibit prominent absorption peaks ≈529 nm. Thus, once formed, they tend to absorb the 517 nm fs laser. Therefore, to mitigate thermal accumulation, higher power densities necessitate a faster scanning speed. These structural transformations were intricately linked to modifications in optical properties, significantly advancing our understanding of perovskite characteristics and their potential applications.

To gain a comprehensive understanding of the nucleation, growth, and optical properties of perovskites processed through the FsLDW technique, we applied the corresponding optimal parameters for (NMA)_2_(FA)_n−1_Pb_n_Br_3n+1_ films with different “n” values (n = 1, n = 2, and n = ∞) to create substrate‐free square periodic lines and concentric circles (Figure S11, Supporting Information). The square structures featured a side length of 1 mm with a spacing of 0.05 mm, while the circular structures had a diameter of 2 mm with a spacing of 0.1 mm. A comparison of images under ambient and UV light revealed a significant increase in fluorescence intensity corresponding to higher “n” values. For an in‐depth analysis of perovskite morphology within the PAN polymer, TEM was employed, as depicted in Figure S12 (Supporting Information). The perovskite (NMA)_2_(FA)_n−1_Pb_n_X_3n+1_ nanoparticles generated through fs laser processing were found to be encapsulated within the polymer matrix. This observation highlights the precision and efficiency of fs laser processing in generating and positioning the perovskite‐polymer hybrid system, significantly enhancing the stability of perovskite in humid environments through in situ growth inside the polymers.

The above results indicate that using an fs laser to directly induce perovskite growth, 3D perovskite requires less energy versus time accumulation compared to low‐dimensional counterparts. This can be attributed to two primary factors: easier nucleation and growth conditions, yet less thermal stability. The easier nucleation and growth in 3D perovskites stem from several factors,^[^ ^38^, ^39^, ^40^ ^]^ including a continuous 3D network that facilitates crystal growth. For low‐dimensional perovskites, which have alternating organic or inorganic layers, encounter more interfacial and energetic barriers. The layered structure of low‐dimensional perovskites demands more energy to overcome these barriers. Additionally, 3D perovskites exhibit lower surface energy, aiding in crystal growth. In contrast, low‐dimensional perovskites, influenced by their organic components, have higher surface energies, requiring more energy for growth.^[^ ^41^, ^42^, ^43^ ^]^ We further validated this by heating transparent precursor films to induce perovskite growth and recording the time required. As shown in Figure S13 (Supporting Information), the NMA, PEA, and AVA systems with different n‐values were subjected to 100 °C to grow perovskites. The results indicate that as the n‐value decreases, the time required for perovskite growth increases. This trend is consistent with the known behavior that 2D perovskites, which possess higher formation energies compared to their 3D counterparts, are more thermodynamically stable and therefore demand more energy and time to form. The structural advantages, lower energy barriers, and reduced surface energy collectively contribute to the superior nucleation and growth of 3D perovskites.

On the other hand, it is widely recognized that low‐dimensional perovskites exhibit superior thermal stability compared to 3D perovskites.^[^ ^44^, ^45^ ^]^ The organic layers present act as insulating barriers, reducing thermal energy transfer to the inorganic layers. This organic‐inorganic composition protects against thermally induced structural changes or degradation. Additionally, the organic‐inorganic interfaces in low‐dimensional perovskites can absorb or dissipate heat, making them less sensitive to thermal fluctuations. These characteristics endow low‐dimensional perovskites with greater resilience in high‐temperature environments.

### 2.3 FsLDW Technology of Complicated Perovskite Patterns

**Figure 4:**
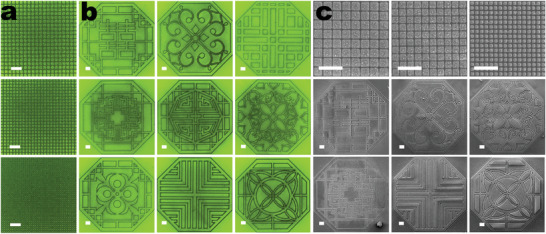
a) Periodic grids with spacing of 10, 8, and 5 µm, and b) geometric quasi‐2D RP perovskite patterns fabricated on glass using FsLDW technology. c) SEM images of the geometric patterns, all the scale bars of (a–c) are 25 µm.

Considering the challenges associated with achieving high‐resolution patterning of perovskites and their significant value in applications such as light‐emitting devices, image sensor arrays, and fluorescent anti‐counterfeiting labels, we further highlight the capability of FsLDW technology in creating intricate, high‐resolution perovskite patterns. We selected (NMA)_2_FAPb_2_Br_7_ as the primary candidate for laser writing processing, given the greater stability of quasi‐2D RP perovskites.^[^ ^46^, ^47^, ^48^ ^]^ Our samples exhibit remarkable long‐term stability in a natural environment at room temperature, maintaining their integrity for over a year (Figure S14, Supporting Information). The PL intensity remained consistent throughout the storage period, with no significant changes observed daily. Additionally, the samples demonstrate water‐proof properties and remained stable for over 72 hours underwater (Figures S15 and S16, Supporting Information). This exceptional stability of our processed perovskite system underscores its potential for future anti‐counterfeiting applications, providing a solid foundation for practical and reliable usage. As illustrated in **Figure** **4**a and Figure S17 (Supporting Information), we present periodic square arrays with pitches of 10, 8, and 5 µm from top to bottom, respectively. Additionally, Figure 4b showcases patterns with diverse window designs. All patterns exhibit uniform fluorescence properties, and their scanning electron microscopy (SEM) images reveal distinct outlines, indicating the successful application of FsLDW technology for precise perovskite patterning (Figure 4c).

### 2.4 Processing of Perovskite Patterns on Different Substrates

**Figure 5:**
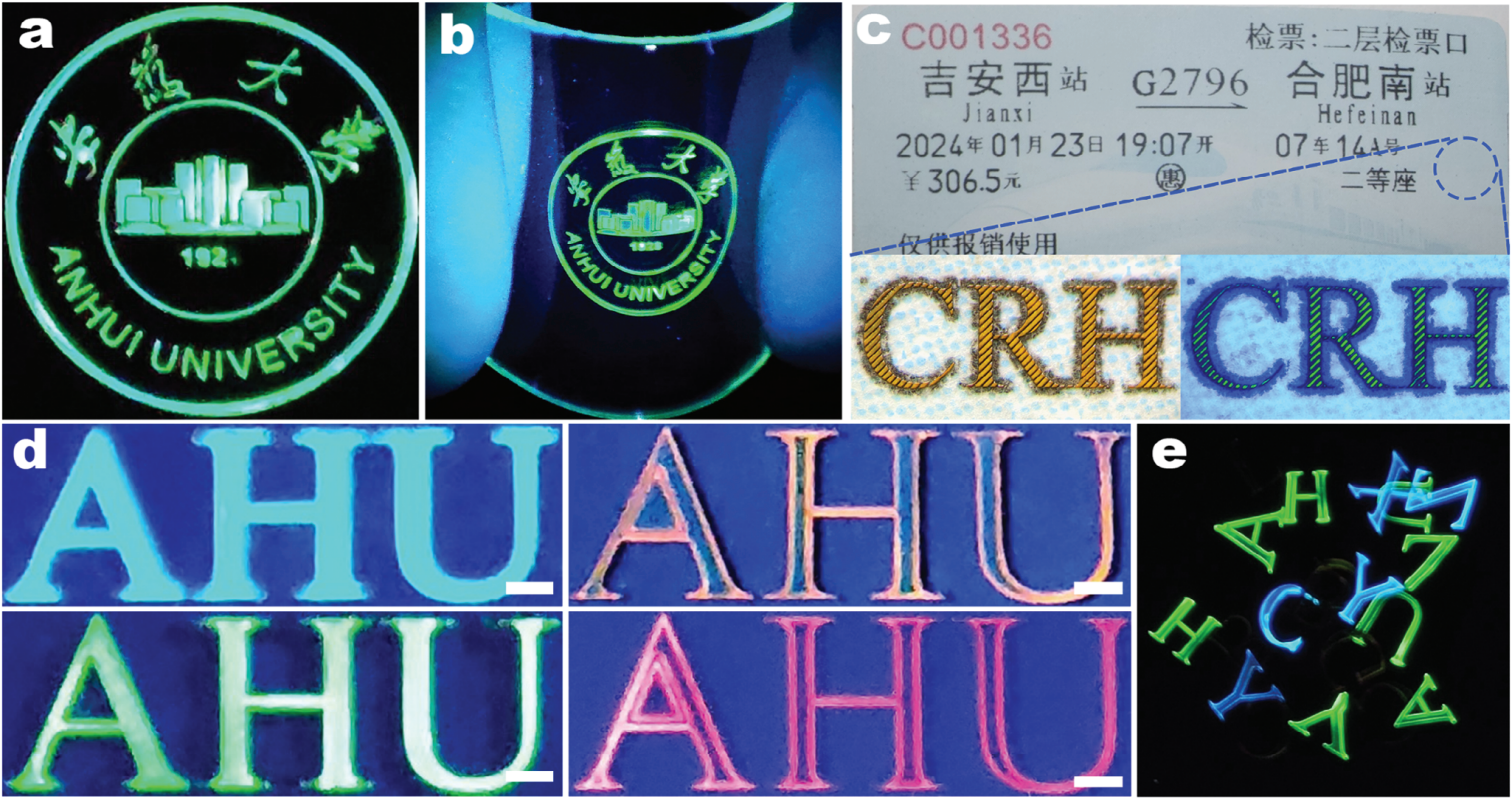
a) An emblem pattern processed from the precursor film on a single‐crystal silicon wafer. b) An emblem pattern processed from the precursor film on a flexible PDMS substrate. c) The pattern representing “CRH” was processed on a ticket of China Railway Highspeed using FsLDW, the blue circle indicates the enlarged area and is displayed under 365 nm UV light. d) The samples were processed under 365 nm UV light after replacing the X in the precursor (NMA)_2_(FA)_n−1_Pb_n_X_3n+1_ with various other halogen elements, with a scale of 300 µm. e)The various scattered letters are presented, which were processed from precursor films without a substrate.

Our technology is versatile and extends to processing on various rigid or flexible substrates for display applications. For instance, we affixed the precursor perovskite/PAN films to silicon wafers or flexible PDMS substrates. The resulting patterns effectively adhere to the silicon or PDMS substrate, as depicted in **Figure** **5**a,b. These findings underscore the potential of FsLDW technology for fabricating perovskite films on flexible substrates for flexible displays. Additionally, we use FsLDW technology to produce perovskite patterns on a ticket of China Railway Highspeed for anti‐counterfeiting. The marked tickets emit green light under UV light (Figure 5c), and due to the protective polymer matrix, these markings can endure for an extended duration.

By substituting the halide element “Br” with “Cl” or “I,” we successfully achieved visible light emission ranging from blue to red within the (NMA)_2_(FA)_n−1_Pb_n_X_3n+1_ perovskite system. As depicted in Figure 5d, the blue‐emitting perovskite film, constructed from (NMA)_2_FAPb_2_Cl_7_ perovskite and PAN polymer, exhibits distinctive blue‐emitting patterns under 365 nm UV light. The yellow‐emitting perovskite film is derived from (NMA)_2_FAPb_2_Br_4_I_3_, while the red‐emitting perovskite originates from (NMA)_2_FAPb_2_BrI_6_. Leveraging the advantage of substrate‐free fabrication, we can create freestanding patterns that emit different colors. Figure 5e and Figure S18 (Supporting Information) illustrate the results of processing English letters as examples, demonstrating that without additional substrate support, the processed letter patterns can be separated and stacked on top of each other. Due to the protective PAN layer, each letter emits light individually and stably, avoiding halogen exchange‐induced color changes.

### 2.5 Application of A_2_(FA)_n−1_Pb_n_X_3n+1_ Film Materials in Anti‐Counterfeiting

**Figure 6:**
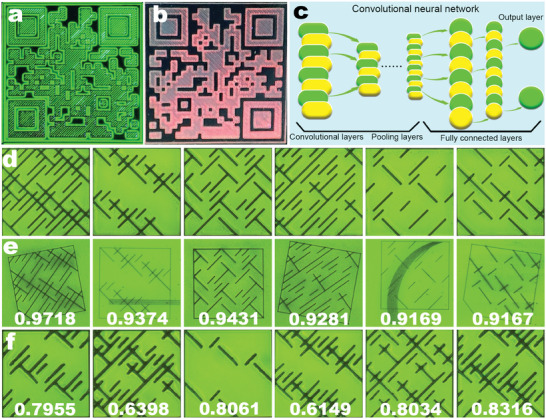
a,b) Rectangular 2D barcodes, measuring 6.75 mm, fabricated from (NMA)_2_FAPb_2_Br_5_I_3_ perovskite films using FsLDW. a) Fabricated on the side facing the laser. b) Fabricated on the side facing the substrate. c) Schematic representation of the convolutional neural network. d) Six anti‐counterfeiting labels representing genuine products in the database. e) Anti‐counterfeiting labels were created on the new film based on the original labels in inset (c). f) The six labels that were not pre‐learned.

By employing a distinctive fabrication technique that involves precise halogen element design and control over the thickness of perovskite precursor films, we can achieve dual‐color emitting patterns simultaneously at the top and bottom through a single FsLDW process. Specifically, we opted to generate a quick‐response (QR) barcode using a film from the mixed‐halide element system (NMA)_2_FAPb_2_Br_5_I_2_ via FsLDW (22.6 kW cm^−2^, 0.25 mm s^−1^). As illustrated in **Figure** **6**a, following processing, the side of the film facing the laser emits green light at 520 nm, while the substrate‐facing side emits red light at 609 nm (Figure S19, Supporting Information). The ability to generate two distinct structures in a single‐step fabrication process is attributed to the intelligent utilization of the asymmetric distribution of halogen elements. Such technology presents a novel approach to anti‐counterfeiting through FsLDW.

To further enhance tamper resistance and elevate the anti‐counterfeiting performance through FsLDW technology, we integrated laser fabrication with deep learning techniques. This involved creating 100 micro‐hole array images, each featuring a unique identifier derived from the first 12 bits of a SHA256 hash value, generated by combining a key with the hash algorithm. The array images, accompanied by rectangular labels, were generated using a specialized micro‐hole array generation program (refer to Figure S20, Supporting Information). In each image, pixels representing odd characters in the identifier were rendered black, while even characters corresponded to white pixels, thus forming distinctive rectangular anti‐counterfeiting labels. The rapid processing capability of FsLDW enabled us to complete the entire procedure within 15 min, achieving an impressive 98% success rate (see Figure S21, Supporting Information).^[^ ^29^, ^49^ ^]^ Our comprehensive deep learning approach involved a convolutional neural network (CNN), comprising convolutional layers, pooling layers, and fully connected layers (refer to Figure 6c). We employed 98 fluorescence images as the database for the deep learning database. To augment the diversity of input data, we pre‐processed the images with random cropping, rotation, horizontal flipping, and normalization. Following training, the model was saved for subsequent label recognition tasks.

Subsequently, we selected six images for AI identification (refer to Figure 6d). Acknowledging the potential challenges faced by anti‐counterfeiting labels, such as damage and contamination, as well as variations in photography angles, we employed FsLDW technology to reprocess these six images (see Figure 6e). This step was designed to simulate real‐world conditions in which anti‐counterfeiting labels might experience contamination, damage, distortion, or changes in orientation during photography. Subsequently, we evaluated the capability of AI to recognize these reprocessed images, noting recognition reliabilities of 0.9718, 0.9374, 0.9431, 0.9281, 0.9169, and 0.9167 for each image, respectively. Following this, we tested the recognition capabilities of AI on six images that were not included in its learning dataset, resulting in recognition reliabilities of 0.7955, 0.6398, 0.8061, 0.6149, 0.8034, and 0.8316 (see Figure 6f). By establishing a threshold of 0.9 and considering confidence levels, we could accurately determine the authenticity of the labels. This process exemplifies the fusion of FsLDW with AI as an innovative strategy for intelligent anti‐counterfeiting technologies.

## 3 Conclusion

In conclusion, this research comprehensively demonstrates the versatility and precision of FsLDW in fabricating intricate perovskite patterns for intelligent anti‐counterfeiting applications. Through meticulous control of fabrication parameters, we successfully created multi‐dimensional (2D, quasi‐2D RP, and 3D) perovskite patterns, providing insights into their nucleation, growth, and optical properties. Our technique, utilizing different halogen element distribution designs, enabled the one‐step laser creation of dual distinct color QR code patterns. These symmetrical QR codes present a unique method for anti‐counterfeiting. Furthermore, the combination of perovskite patterns with deep learning technology for label recognition enhanced the reliability and tamper resistance of anti‐counterfeiting marks. This integrated approach introduces a novel perspective for future anti‐counterfeiting technologies, making security features more secure and dependable. Our research not only provides new insights into the application of FsLDW technology in multi‐dimensional perovskite systems but also offers effective solutions for improving the credibility and anti‐counterfeiting performance of security measures. These outcomes have a significant impact on areas such as photonics, and ticket security, paving the way for practical applications of perovskites in high‐resolution displays, image sensor arrays, and other optoelectronic devices.

## 4 Experimental Section

### Chemicals Used in the Experiments

Formamidinium acetate (FAA, 99%), 1‐naphthylamine (98%), 5‐aminovaleric acid hydrobromide (AVABr, 99%), 2‐phenylethylamine hydrobromide (PEABr, 99%), 4‐phenylbutan‐1‐amine hydrobromide (PhBABr, 99%), 1‐methyl‐4,5‐dihydro‐1h‐imidazol‐2‐amine hydrobromide (MDIBr, 99%), and alpha‐amino‐gamma‐butyrolactone hydrobromide (ABLBr, 99%), hydrobromic acid (HBr, 40%), hydroiodic acid (HI, 55%), lead chloride (PbCl_2_, 99%), lead bromide (PbBr_2_, 99%), lead iodide (PbI_2_, 99%), methanol (99.9%), n‐hexane (99%), dichloromethane (99.9%), tetrahydrofuran (THF, 99%), N, N‐Dimethylformamide (DMF, 99%), and polyacrylonitrile (PAN, 250000 Mw) were purchased from Macklin. Hydrochloric acid (HCl, 38%) and anhydrous ether (99.0%) were obtained from Sinopharm Chemical Reagent Co Ltd.

### Synthesis Process of NMAX (X = Cl, Br, I)

15.2 mmol of HX and 12.72 mmol of 1‐naphthylamine were slowly added to 50 mL of THF at 0 °C and stirred for 2 h. The reaction mixture was evaporated at 50 °C for 24 h to obtain a precipitate. The precipitate was washed three times with a THF: CH_2_Cl_2_ (3:1) solution. Finally, the precipitate was vacuum‐dried for 24 h.

### Synthesis Steps for FAX (X = Cl, Br, I)

At 0 °C, 0.2 mol of formamidinium acetate was dissolved in 30 mL of anhydrous methanol with vigorous stirring until complete dissolution. 50 mL of HX was slowly added to the solution and stirred  for at least 4 h. The solution was evaporated using a rotary evaporator at 60 °C until the solvent was completely removed, leaving behind solid material. The solid material was dissolved in anhydrous ethanol mixed with n‐hexane to promote crystallization. The upper layer of the solution was discarded and the remaining solution was recrystallized using ether. Finally, white crystals were collected and vacuum dried at 60 °C for 24 h.

### Preparation of A_2_(FA)_n−1_Pb_n_X_3n+1_ Precursor Solution

For n = 1: 1.48 mmol of NMAX and 0.74 mmol of PbX_2_ were dissovled in 5 mL of DMF, then stirred for 2 h. For n = 2: 0.37 mmol of FAX, 0.74 mmol of NMAX, and 0.74 mmol of PbX_2_ were dissovled in 5 mL of DMF, then stirred for 2 h. For n = ∞: 0.74 mmol of FAX and 0.74 mmol of PbX_2_ were dissovled in 5 mL of DMF, then stirred for 2 h. (NMA)_2_(FA)_n−1_Pb_n_(AB)_3n+1_‐type perovskites can be obtained by dissolving the corresponding (NMA)_2_(FA)_n−1_Pb_n_X_3n+1_ precursors in DMF in the proportion of halide elements.

For the precursor solution of A_2_(FA)_n−1_Pb_n_X_3n+1_ with other amines, the procedure was similar to that used for NMA. It simply required substituting NMA with the corresponding amount of the desired long‐chain amine.

### Preparation of A_2_(FA)_n−1_Pb_n_X_3n+1_ Precursor Films

2 g of PAN was dissolved in 20 mL of DMF to preare PAN/DMF solution. The precursor perovskite solution was mixed with the PAN/DMF solution in a 1:4 volume ratio. After stirring for 2 h, the mixture was dropped onto a substrate and dried at 50 °C. This process resulted in transparent precursor films.

### The fs Laser Direct Writing Process

A high‐power, high‐energy fs laser system (MONACO 517‐40‐30) was used to emit laser beams with a central wavelength of 517 nm, a pulse width of 350 fs, and a repetition rate of 750 KHz. The laser output power was controlled by program control software. The pulsed laser was focused using a high numerical aperture objective lens (NA = 0.8, 100x & NA = 0.45, 10x, OLYMPUS). During pattern fabrication, a displacement platform (Newport, Model XPS‐D) and a shutter switch were used in combination.

### Materials Characterization

Fluorescence images of the perovskite pattern morphology were characterized using a microscope (BX53MTRF‐S, OLYMPUS). Scanning Electron Microscope (SEM) images of the patterned perovskite thin films were captured using the SEM equipment (Regulus 8230, Hitachi). Transmission Electron Microscope (TEM) images were obtained using the TEM instrument (JEM‐F200, JEOL). UV–vis absorption spectra of the patterned perovskite thin films were recorded using a UV–vis spectrophotometer (UV‐2600i, SHIMADZU). The PL testing system consisted of a laser with a wavelength range of 405 nm (LASERLAND), a microscope (YM710R, YUESCOPE), and a spectrometer (QE65Pro, OCEAN INSIGHT). XRD testing of the samples was carried out using a multifunctional X‐ray diffractometer (Empyrean S3, Malvern Panalytical).

### Convolutional Neural Network Model

Five Convolutional Layers were used to extract features from the anti‐counterfeiting label images. The first layer used 96 11 & × & 11 convolutional kernels, the second layer used 256 5 & × & 5 convolutional kernels, the third and fourth layers used 3843 & × & 3 convolutional kernels, and the fifth layer used 256 3 & × & 3 convolutional kernels. Each convolutional layer was followed by a ReLU activation function, forming five ReLU activation layers in total. Additionally, three 3 × 3 max‐pooling layers were placed after the first, second, and fifth ReLU activation layers. To prevent overfitting during training, three fully connected layers were included, with dropout applied. The first and second fully connected layers each contained 512 nodes, followed by LeakyReLU activation functions and a dropout rate of 0.5.

### Deep Learning

The deep learning portion of the program was written in Python and utilized the PyTorch library to build and train convolutional neural network models. The program reads image data directly from the file system for input and output, and it saves the trained models back to the file system. The entire training process for the deep learning models took ≈1 hour. The deep learning computer was equipped with the following specifications: CPU (Intel Core i7‐12700K), GPU (NVIDIA GeForce GTX 3060), RAM (16 GB), and HDD (2 TB). The computer had a rated power consumption of 800 W h^−1^.

## Conflict of Interest

The authors declare no conflict of interest.

## Supporting information

Supporting Information

## Data Availability

The data that support the findings of this study are available from the corresponding author upon reasonable request.
