# Supplementary material for: Laser Fabrication of Multi‐Dimensional Perovskite Patterns with Intelligent Anti‐Counterfeiting Applications
Source: Adv Sci (Weinh). 2024 Aug 9;11(42):2309862. doi: 10.1002/advs.202309862 (PMC11558149; doi:10.1002/advs.202309862)
Supplement: Supplementary file 1 — Supporting Information [file ADVS-11-2309862-s001.docx]

**Supporting Information**

**Femtosecond laser fabrication of multi-dimensional perovskite patterns with intelligent anti-counterfeiting applications**

Xiangyu Xu^1,2,3^, Shoufang Liu^1,2,3^, Yan Kuai^2,3^, Yuxuan Jiang^1^, Zhijia Hu^1,2,3^, Benli Yu^1,2,3*^, Siqi Li^1,2,3*^

1. School of Physics and Optoelectronic Engineering, Anhui University, Hefei, 230601, Anhui, P. R. China

2. Information Materials and Intelligent Sensing Laboratory of Anhui Province, Anhui University, Hefei, 230601, Anhui, P. R. China

3. Key Laboratory of Opto-Electronic Information Acquisition and Manipulation of Ministry of Education, Anhui University, Hefei, 230601, Anhui, P. R. China


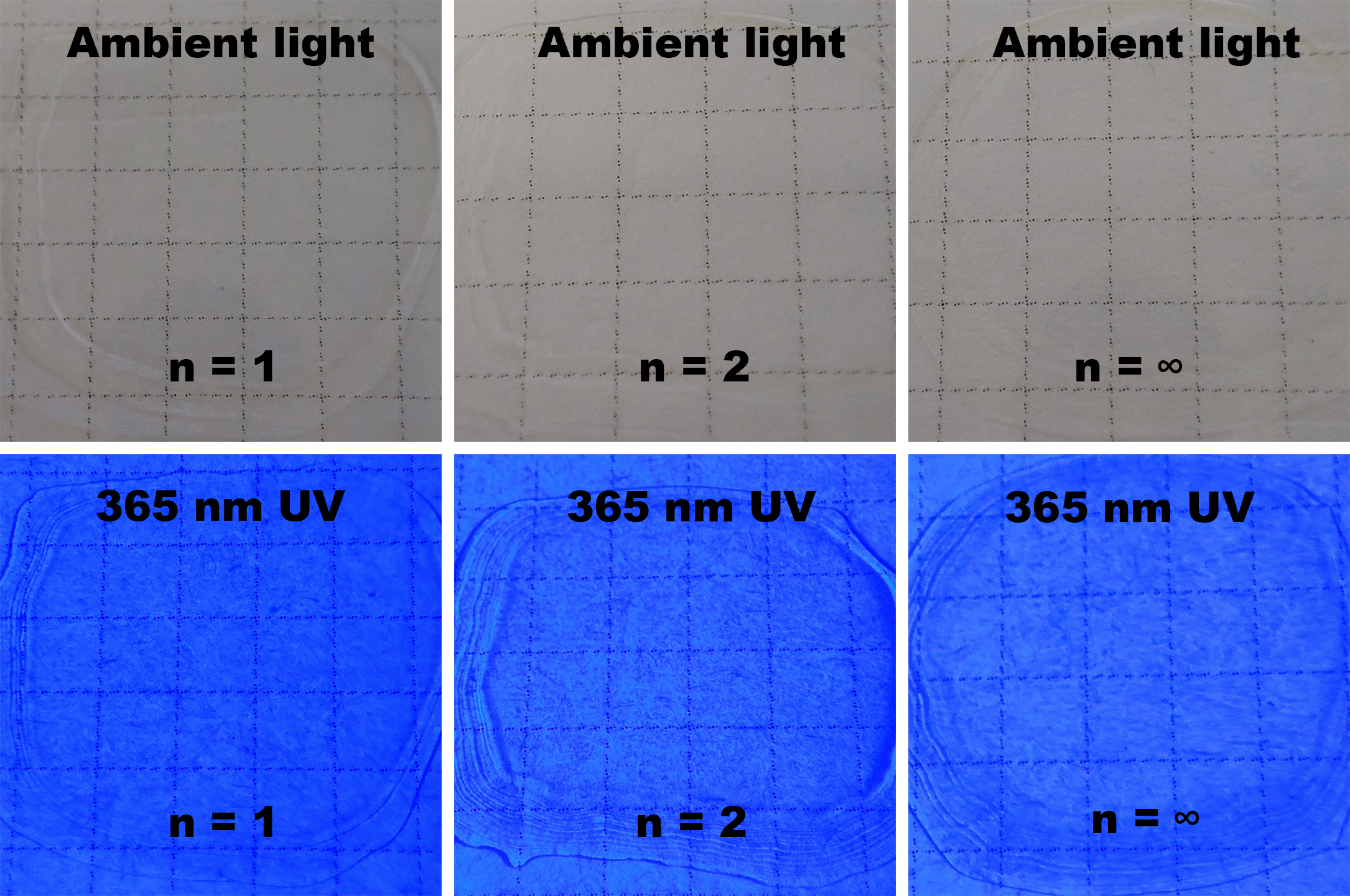


**Figure S1** Images of transparent precursor films for n=1, n=2, and n=∞ under ambient light and 365 nm UV light (NMABr were selected as they represent example)


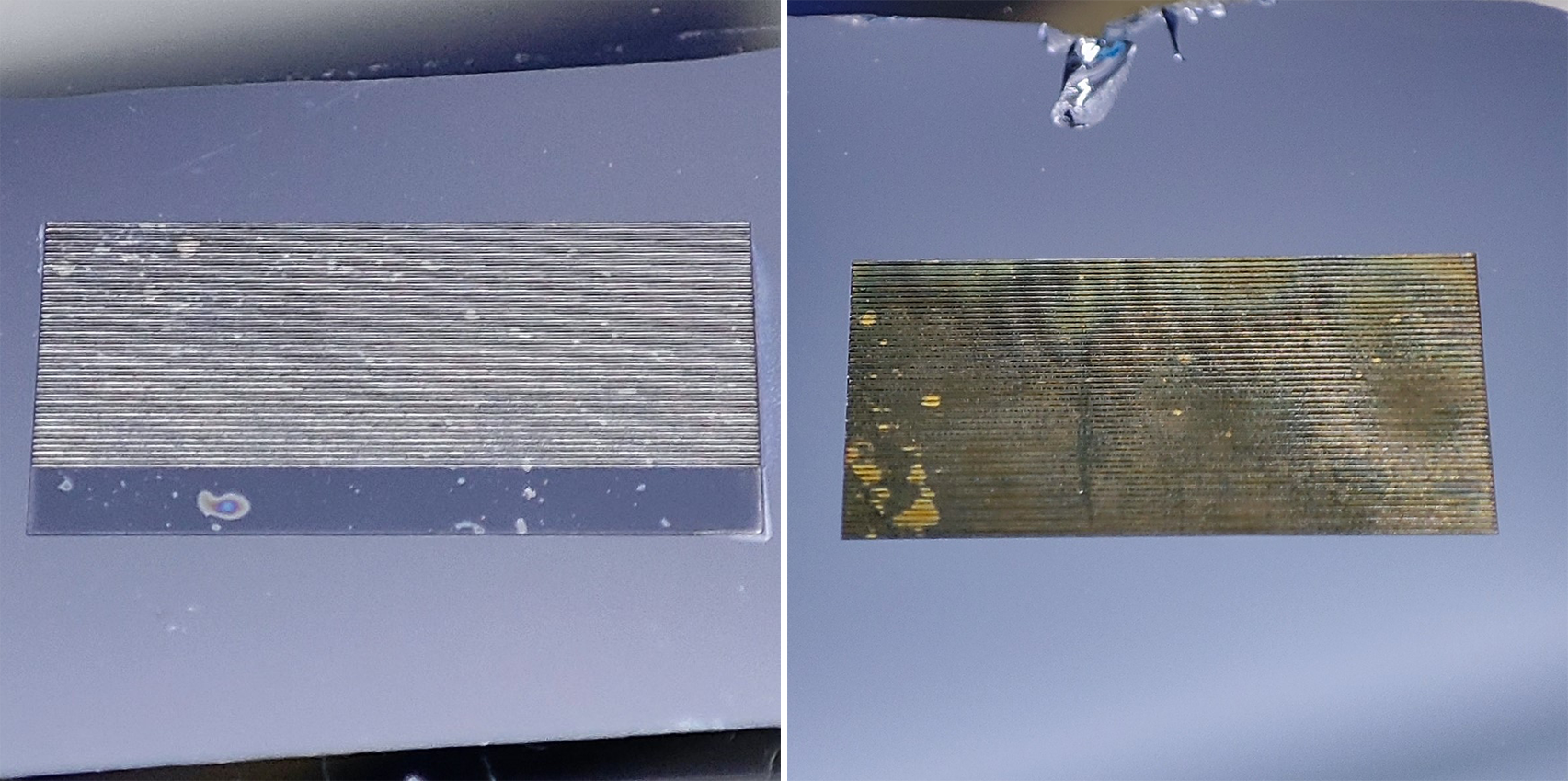


**Figure S2** The sample for XRD measurements on single-crystal silicon wafers.

To facilitate X-ray diffraction (XRD) testing, we adhered transparent films onto single-crystal silicon wafers and created large rectangular areas (10 mm × 5 mm, d=0.1 mm) using FsLDW for testing).


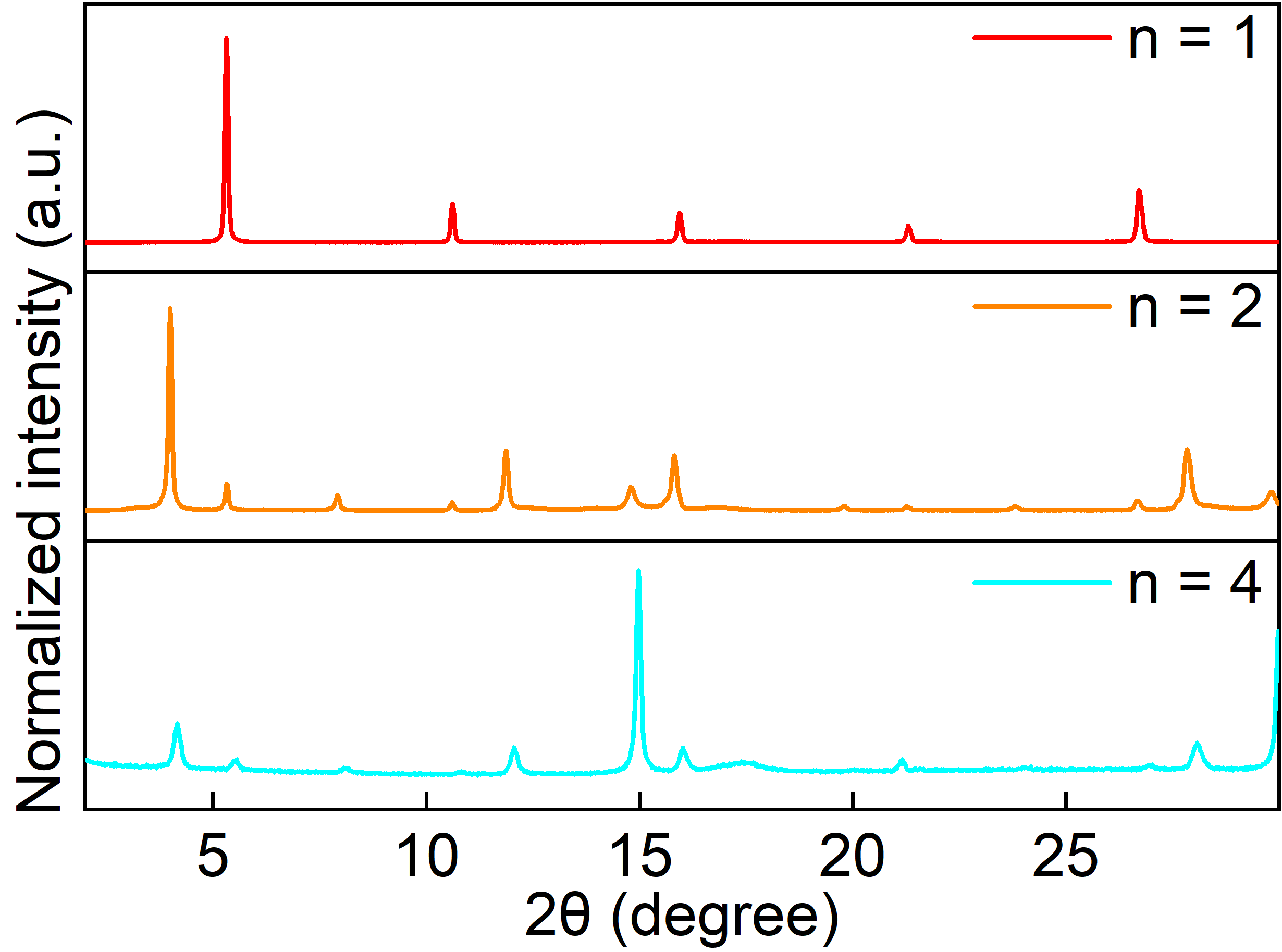


**Figure S3** The XRD patterns of (PEA)_2_(FA)_n-1_Pb_n_Br_3n+1_ films, n=1, n=2, and n=4.


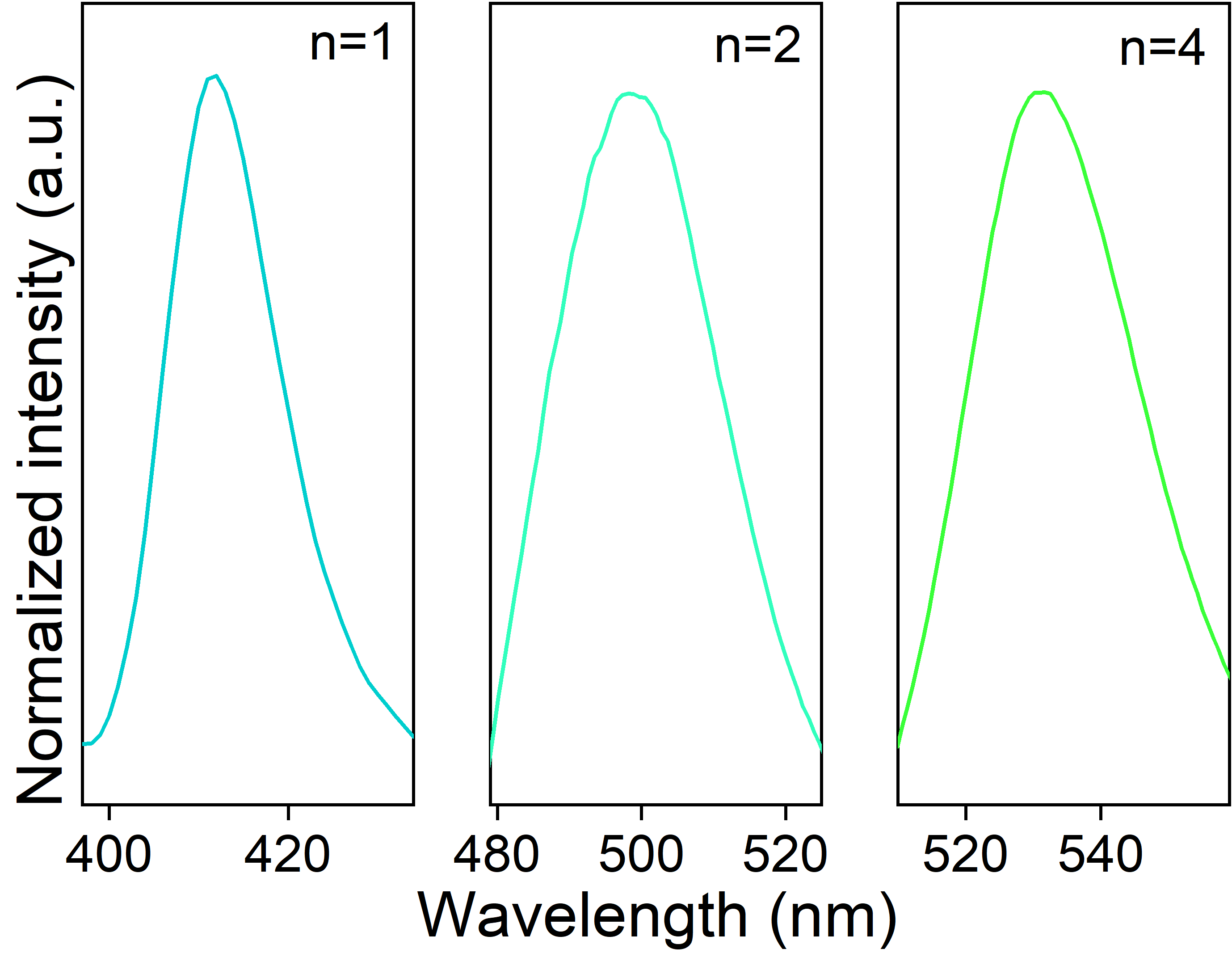


**Figure S4** The PL intensities of (AVA)_2_(FA)_n-1_Pb_n_Br_3n+1_ films.


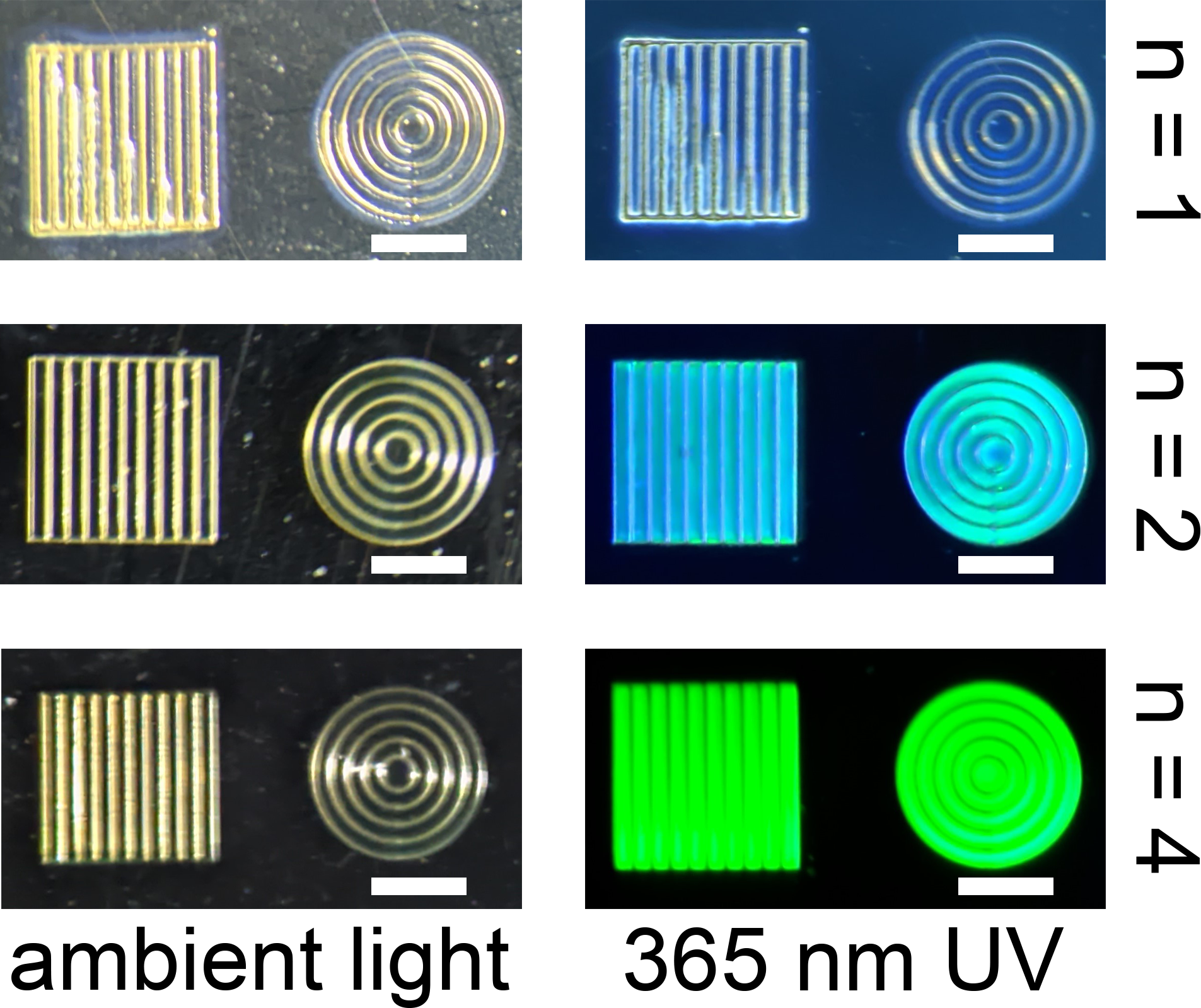


**Figure S5** The pattern of (PEA)_2_(FA)_n-1_Pb_n_Br_3n+1_ films with different "n" values, scale bar is 5 mm.


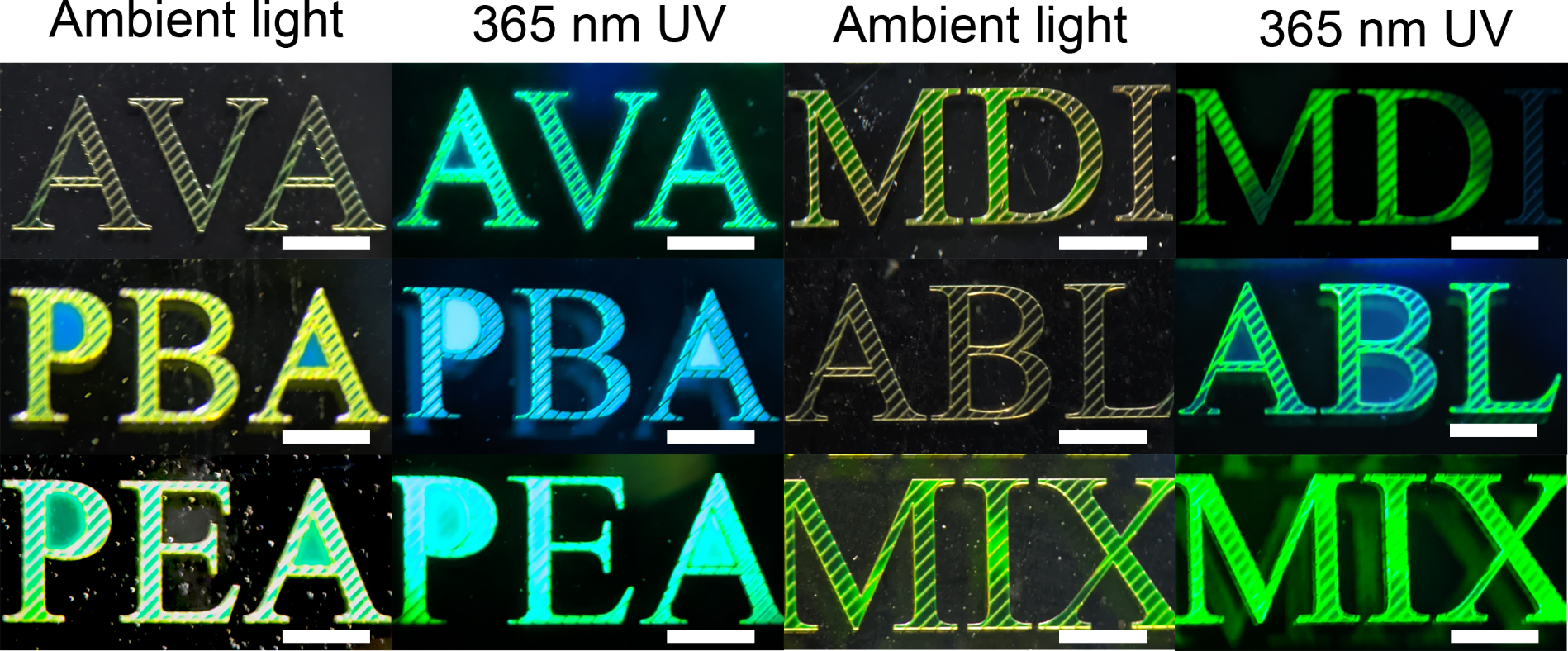


**Figure S6** The A_2_FA_n−1_Pb_n_Br_3n+1_(A = AVA, PEA, PBA, MDI, ABL, NMA, and PEA mixed system) under ambient light and 365nm UV light, scale bar is 1 mm.


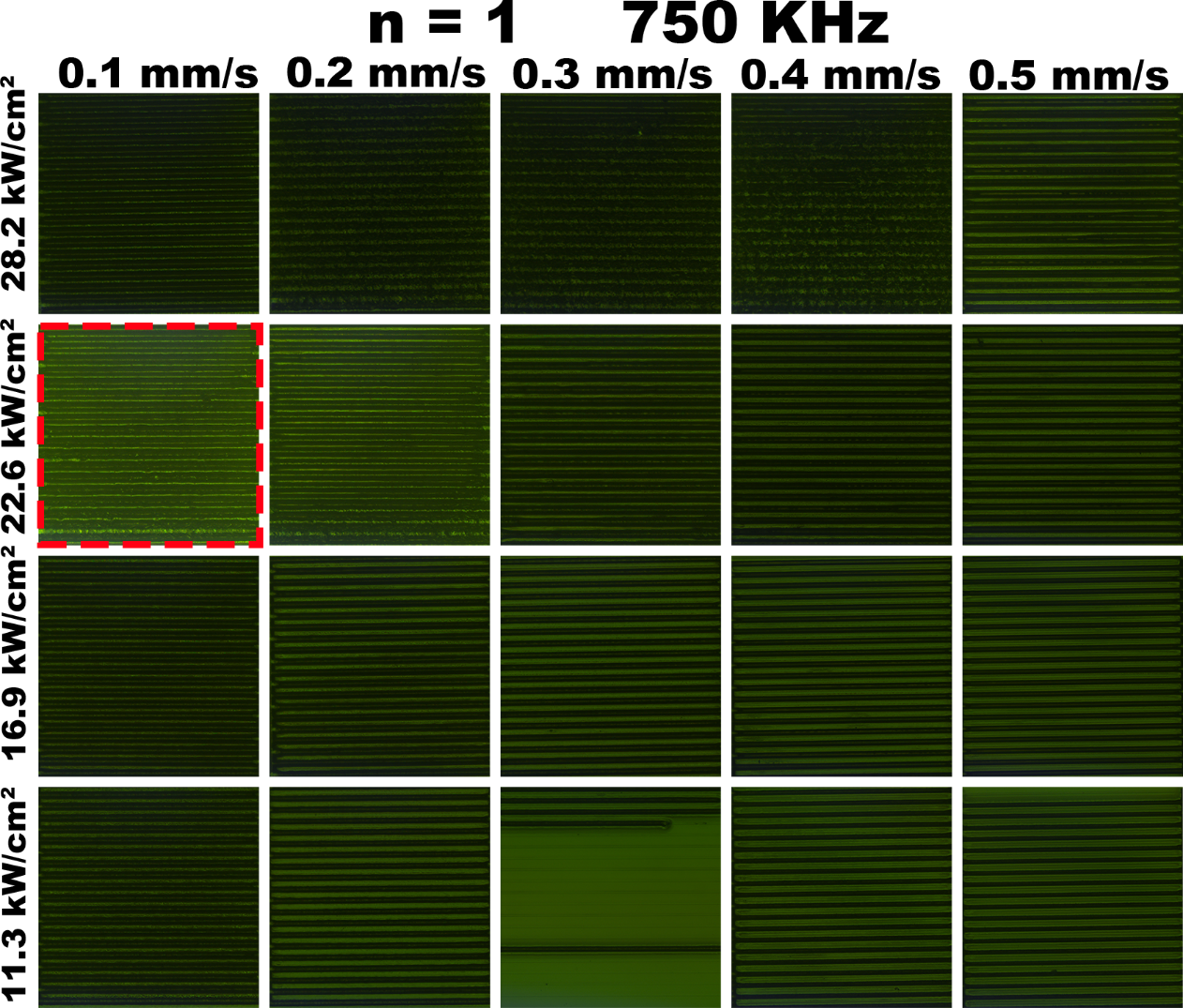


**Figure S7** The 1×1 mm squares of 2D perovskites processed with femtosecond laser at different laser powers and scanning speeds under a repetition rate of 750 kHz.


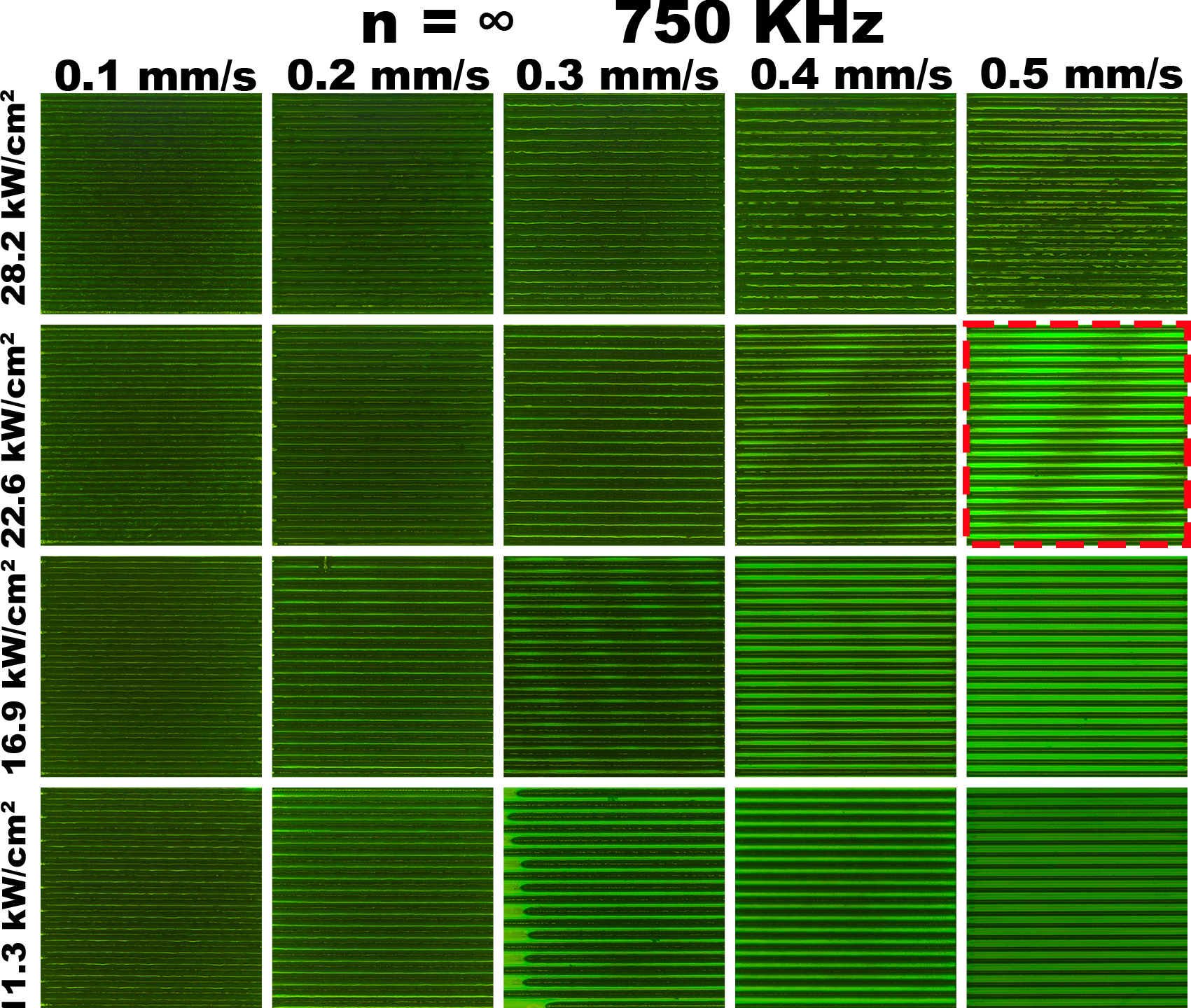


**Figure S8** The 1×1 mm squares of 3D perovskites processed with femtosecond laser at different laser powers and scanning speeds under a repetition rate of 750 kHz.

To justify our choice of the particular repetition rate and energy as favorable for perovskite growth, we defined parameter A as the ratio of power density (kW/cm^2^) to scanning speed (mm/s). Through dimensional analysis, we determined that the unit of A is (J/m^3^), which we interpret as the amount of heat provided per unit volume by the laser. We calculated A for various previously explored processing parameters, yielding the following values:

11.3 kW/cm^2^ / 0.5 mm/s = 22.6 A

16.9 kW/cm^2^ / 0.5 mm/s = 33.8 A

22.6 kW/cm^2^ / 0.5 mm/s = 45.2 A

28.2 kW/cm^2^ / 0.5 mm/s = 56.4 A

……

We then recorded the luminescence intensity of the perovskites fabricated under these conditions and plotted the luminescence intensity against A. By fitting a nonlinear curve to these data points, as shown in **Figure S9**, we observed a clear relationship.


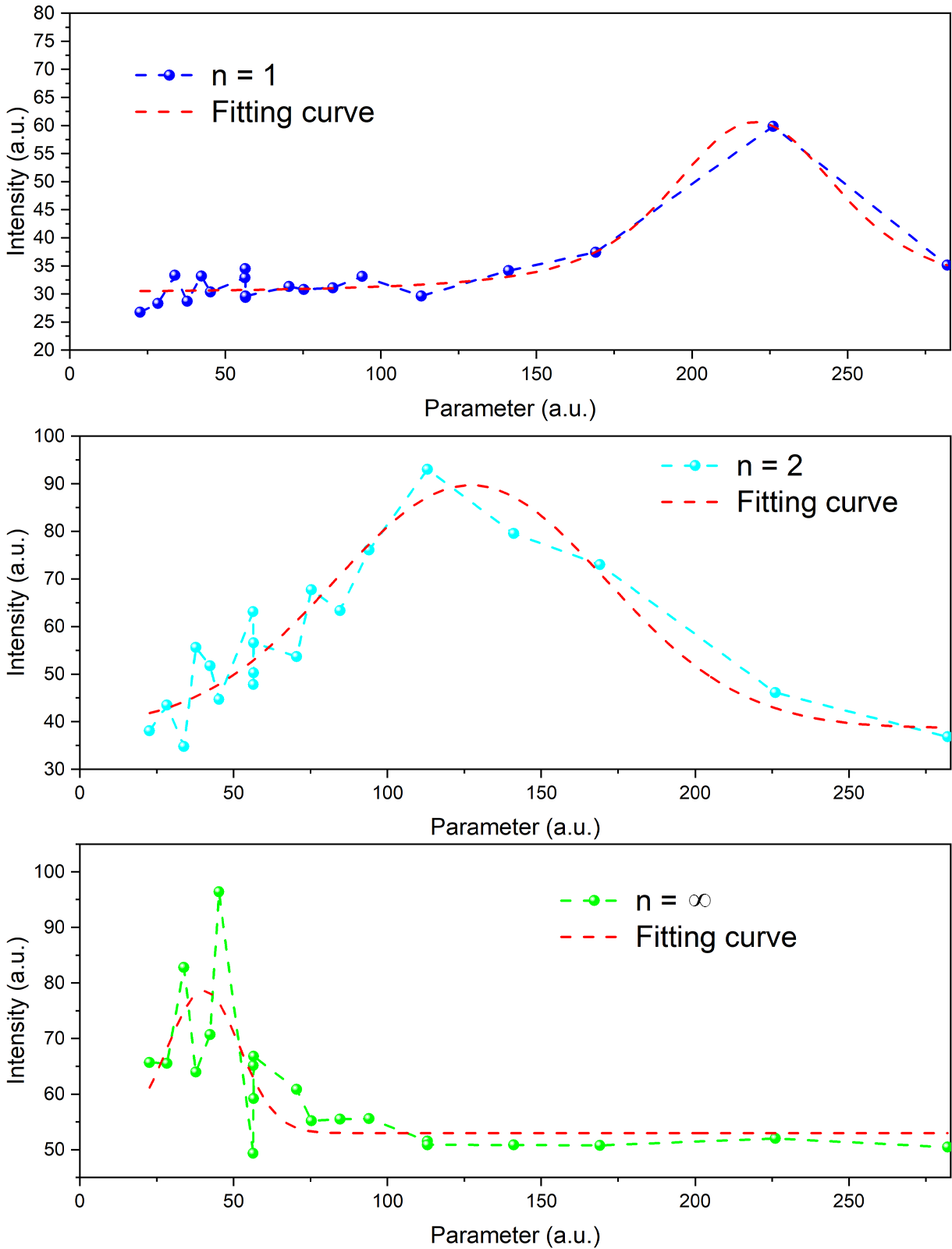


**Figure S9** the luminescence intensity of the perovskites fabricated under these conditions and plots the luminescence intensity against A


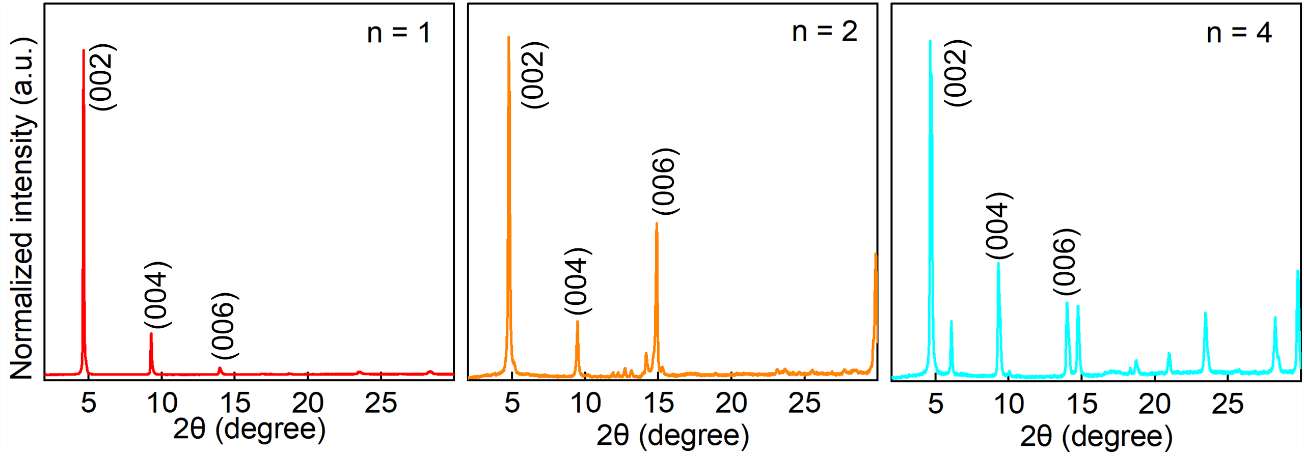


**Figure S10** The XRD patterns of (NMA)_2_(FA)_n-1_Pb_n_Br_3n+1_ films, n=1, n=2, n=4.


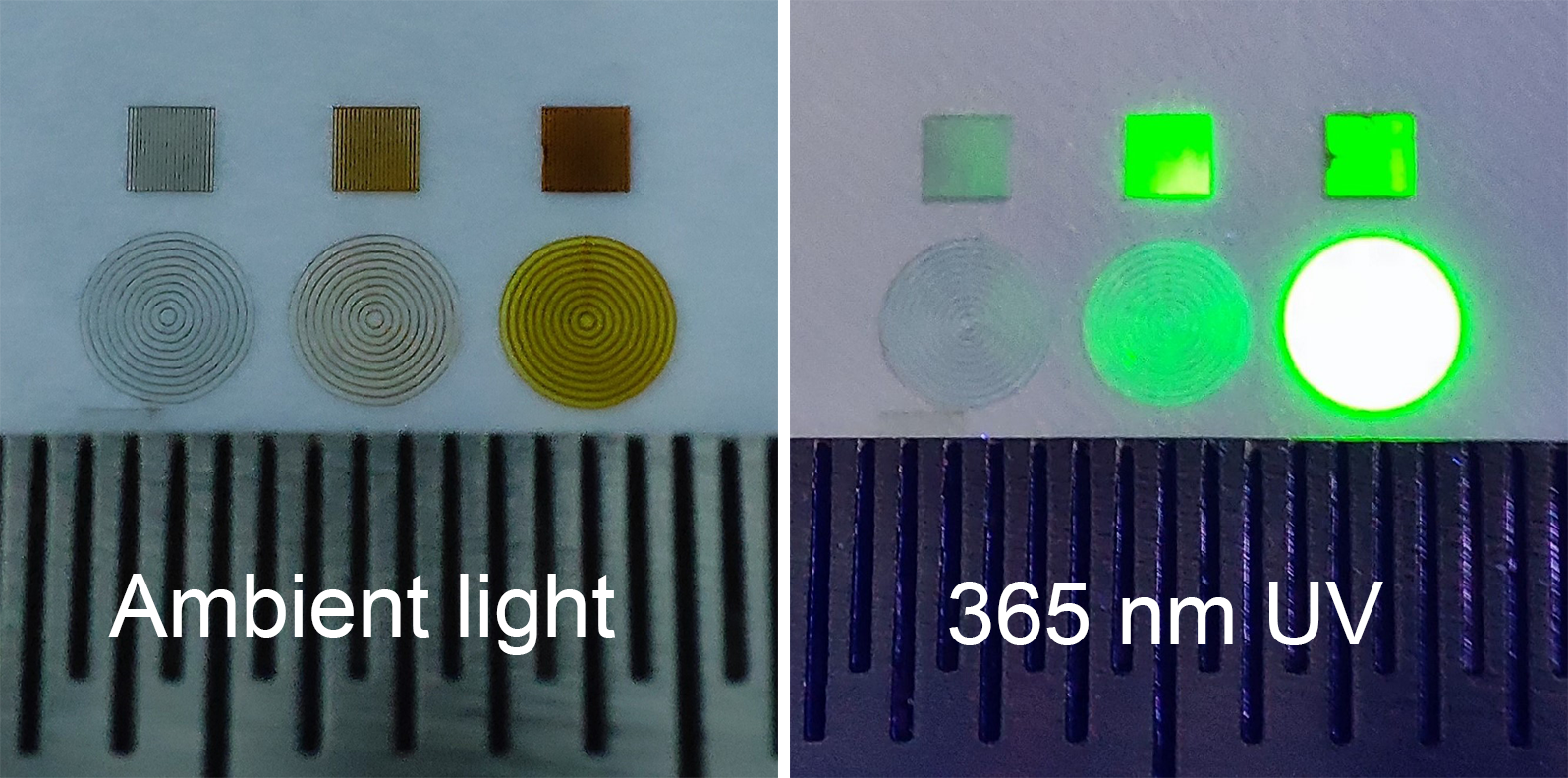


**Figure S11** The pattern of NMA_2_(FA)_n-1_Pb_n_Br_3n+1_ with n= 1, 2, ∞.


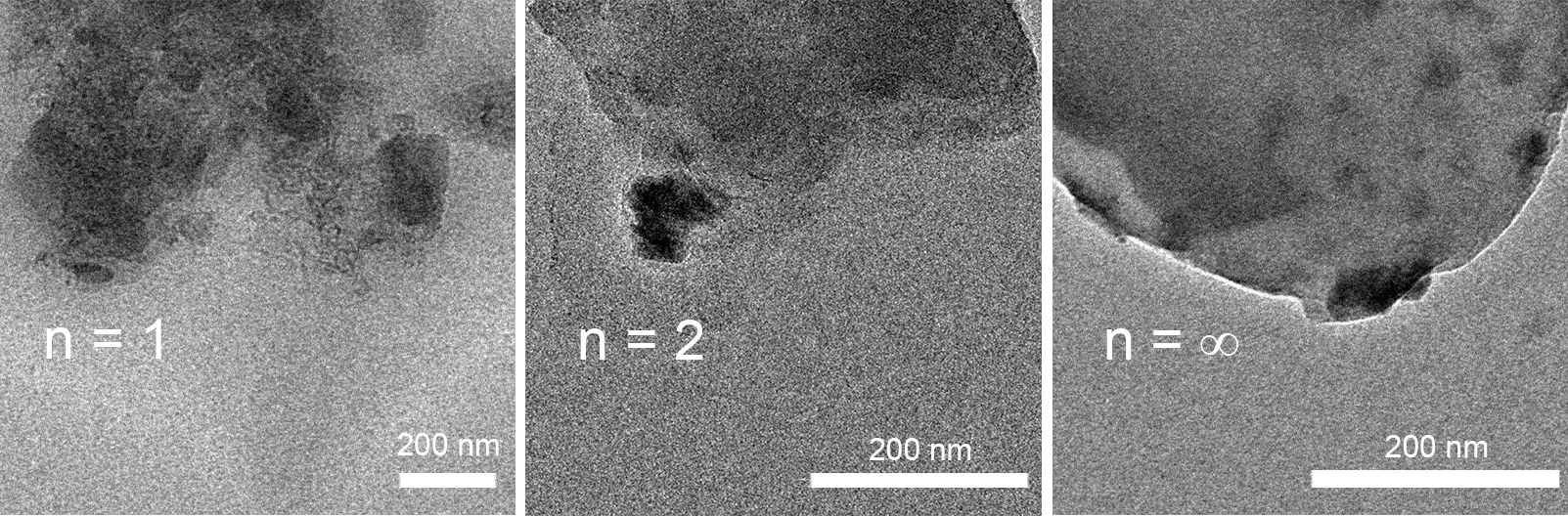


**Figure S12** TEM images of (NMA)_2_(FA)_n-1_Pb_n_Br_3n+1_ films.


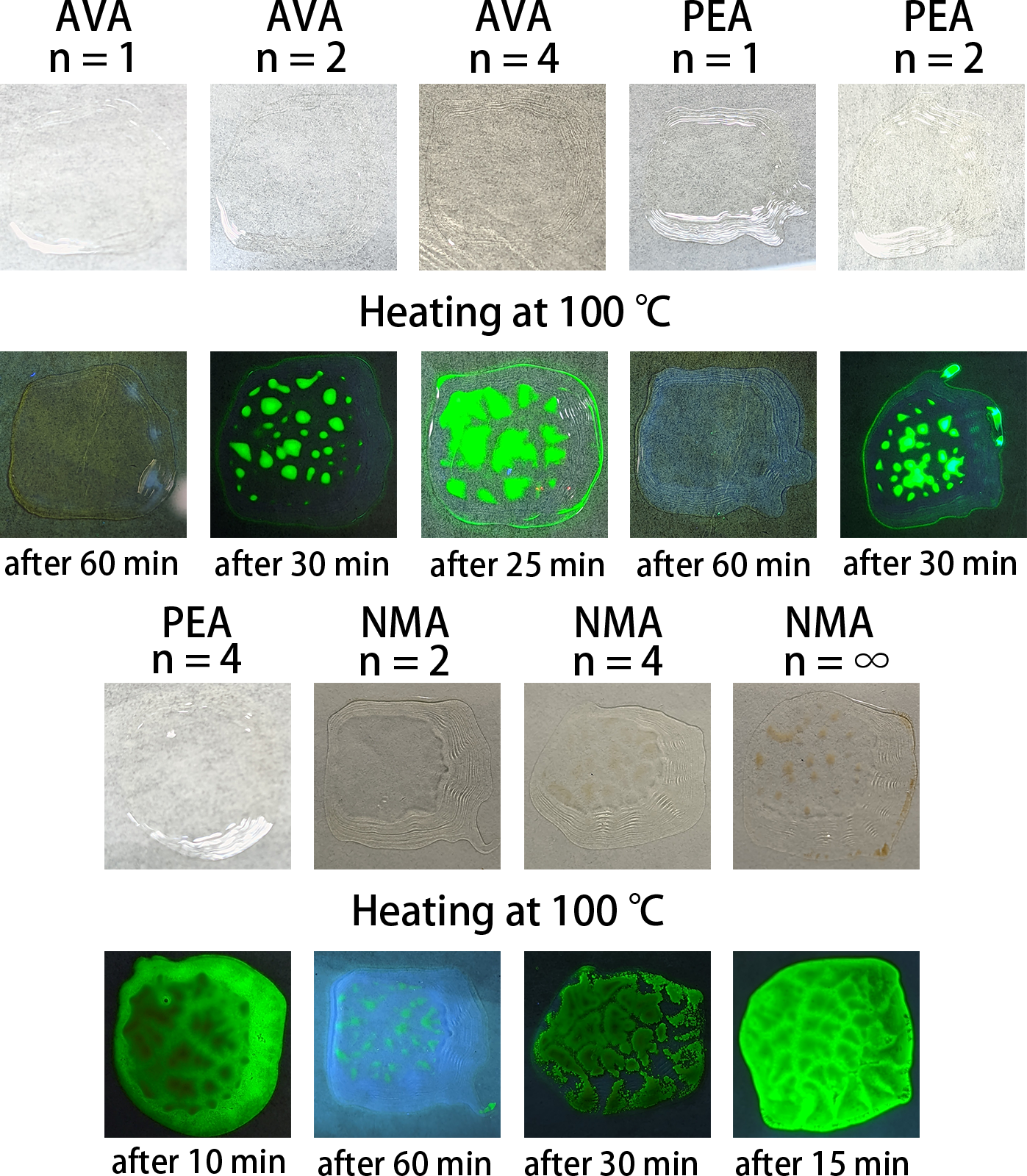


**Figure S13** The A_2_(FA)_n-1_Pb_n_Br_3n+1_(NMA, PEA, AVA) systems with different n-values were subjected to 100°C to grow perovskites.


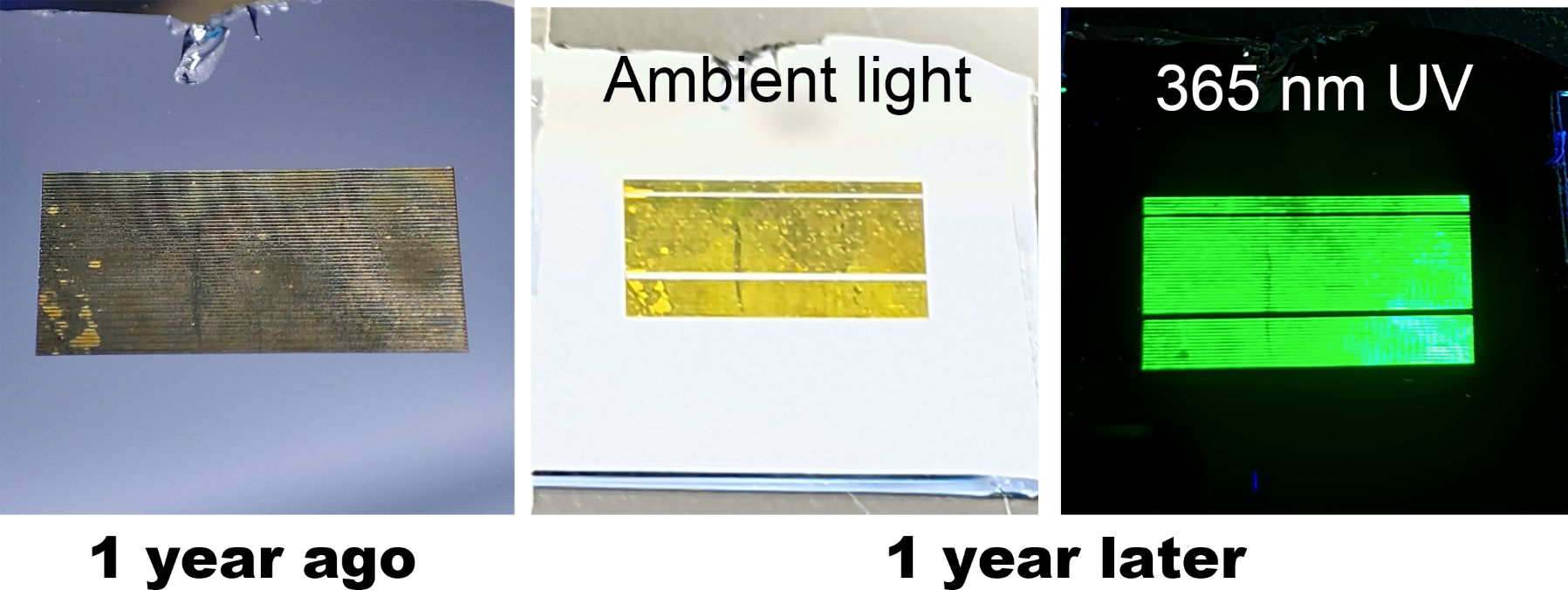


**Figure S14** The samples after one year of storage(right), The samples one year ago (left)


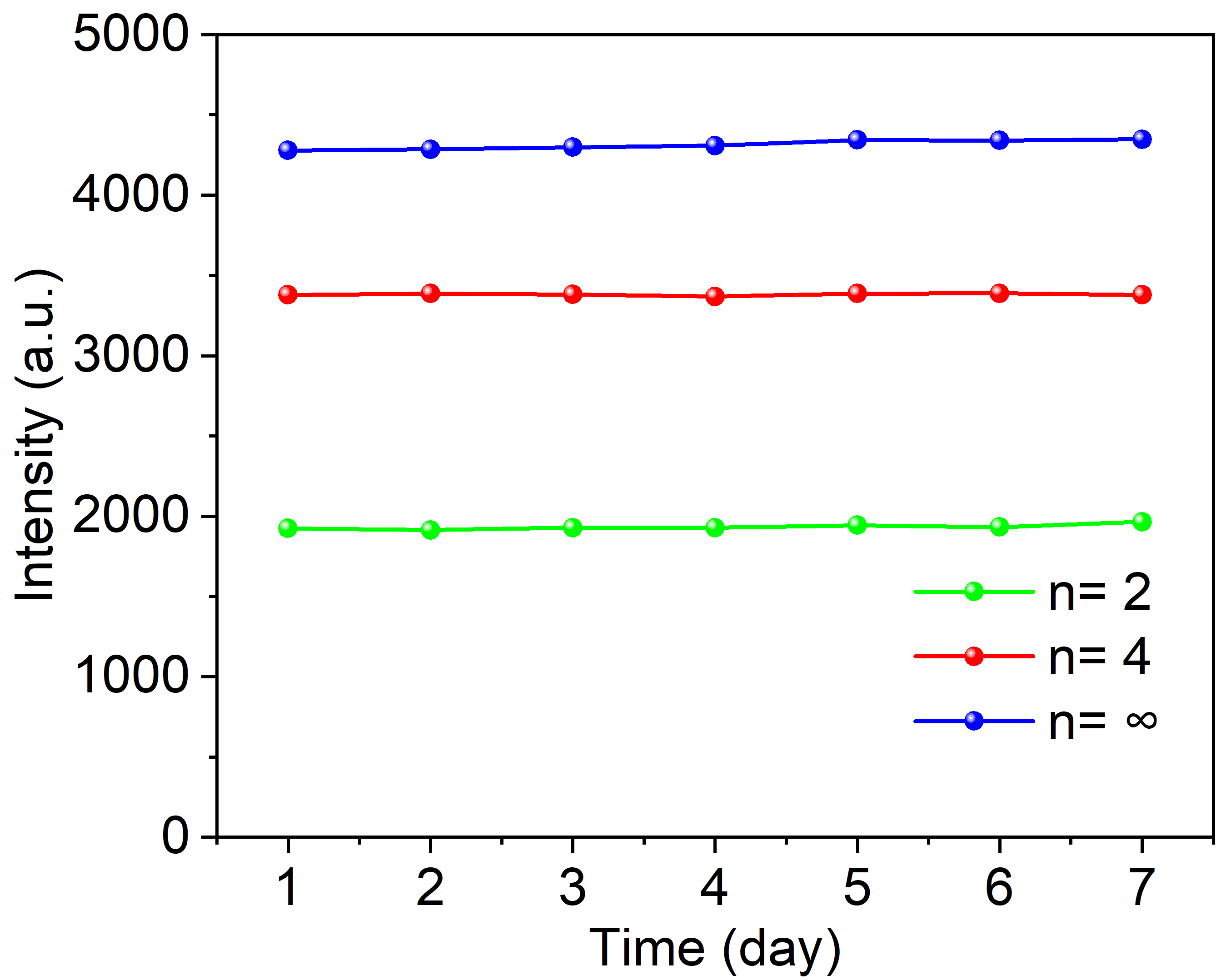


**Figure S15** PL peak intensity of the sample in the air for 7 days


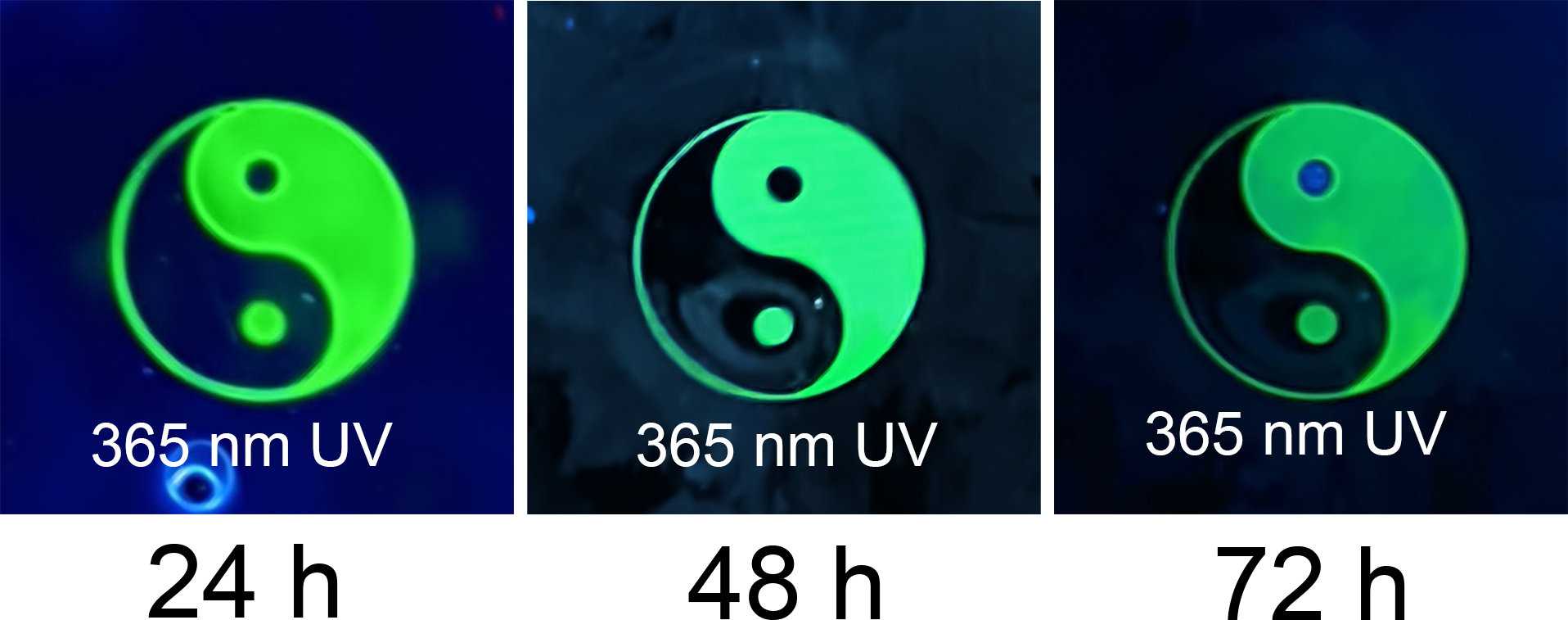


**Figure S16** The samples were underwater for 72 hours


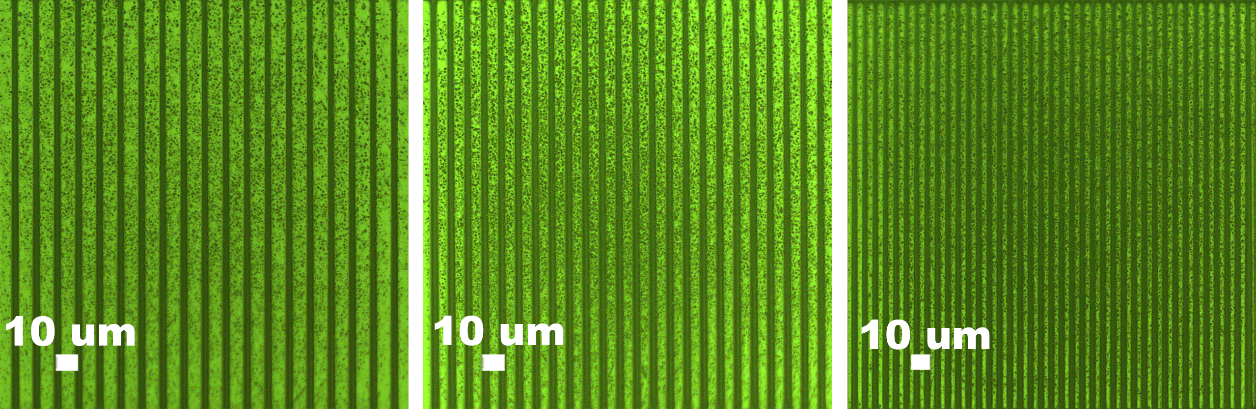


**Figure S17** Periodic straight lines with spacings of 10 µm, 8 µm, and 5 µm from left to right.


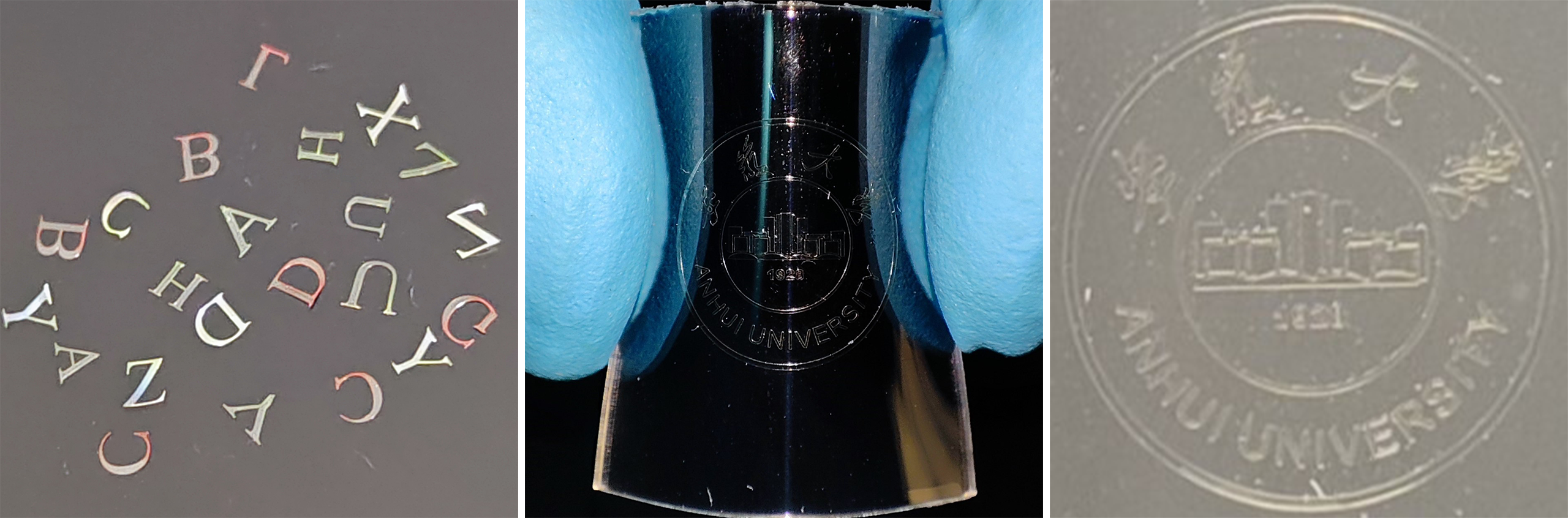


**Figure S18** The patterns after FsLDW processing on bare (no substrate), flexible PDMS substrate, and rigid single-crystal silicon wafer substrate under ambient light.


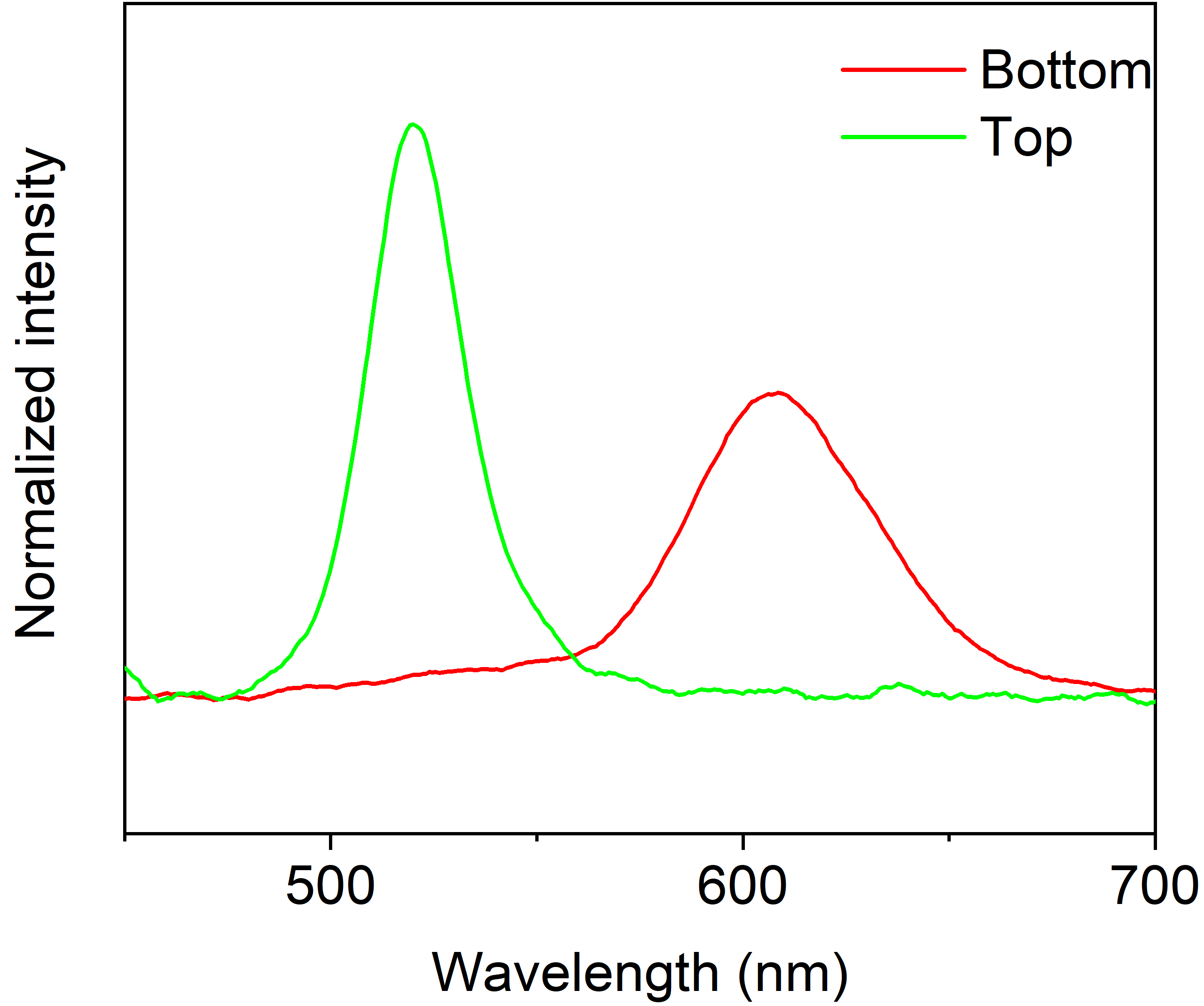


**Figure S19** The QR codes were subjected to 400 nm UV, and the spectra were observed from two perspectives: one from the side facing the laser and the other from the side facing the substrate.


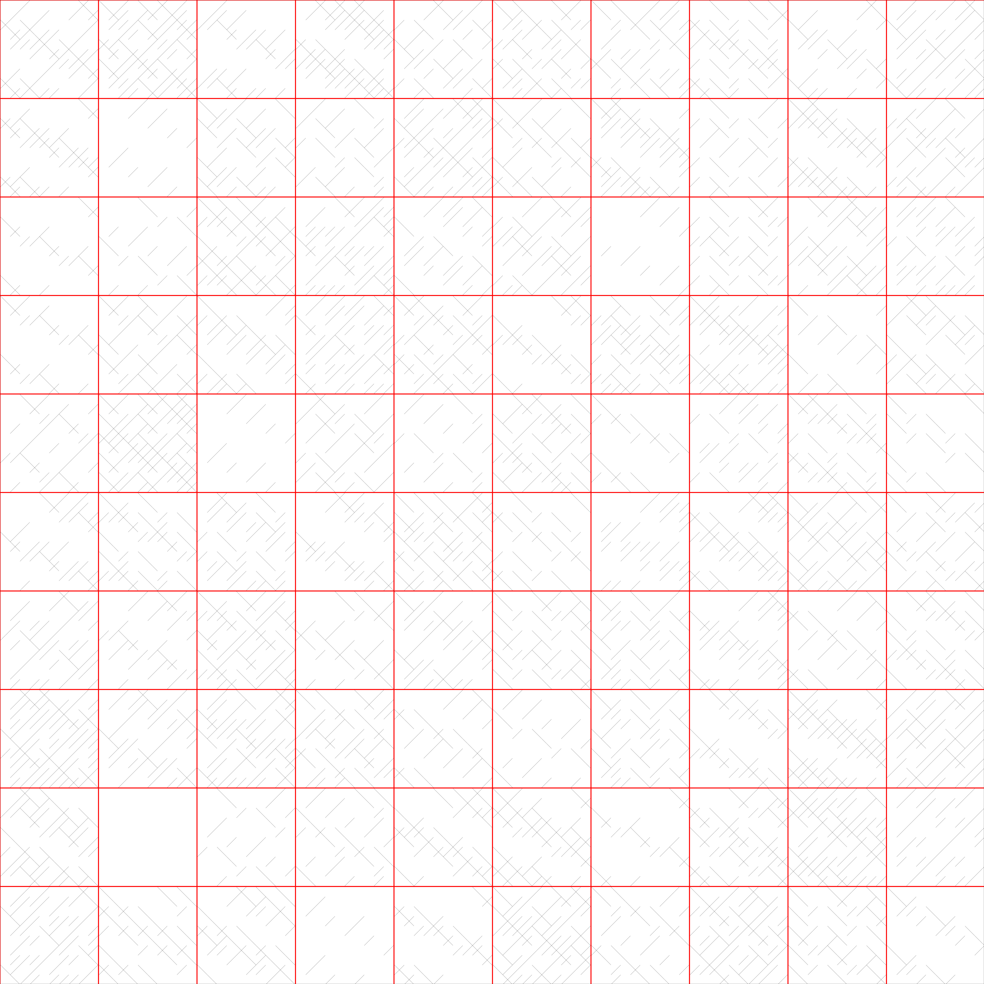


**Figure S20** The 100 micro-aperture array anti-counterfeit labels generated by the program.


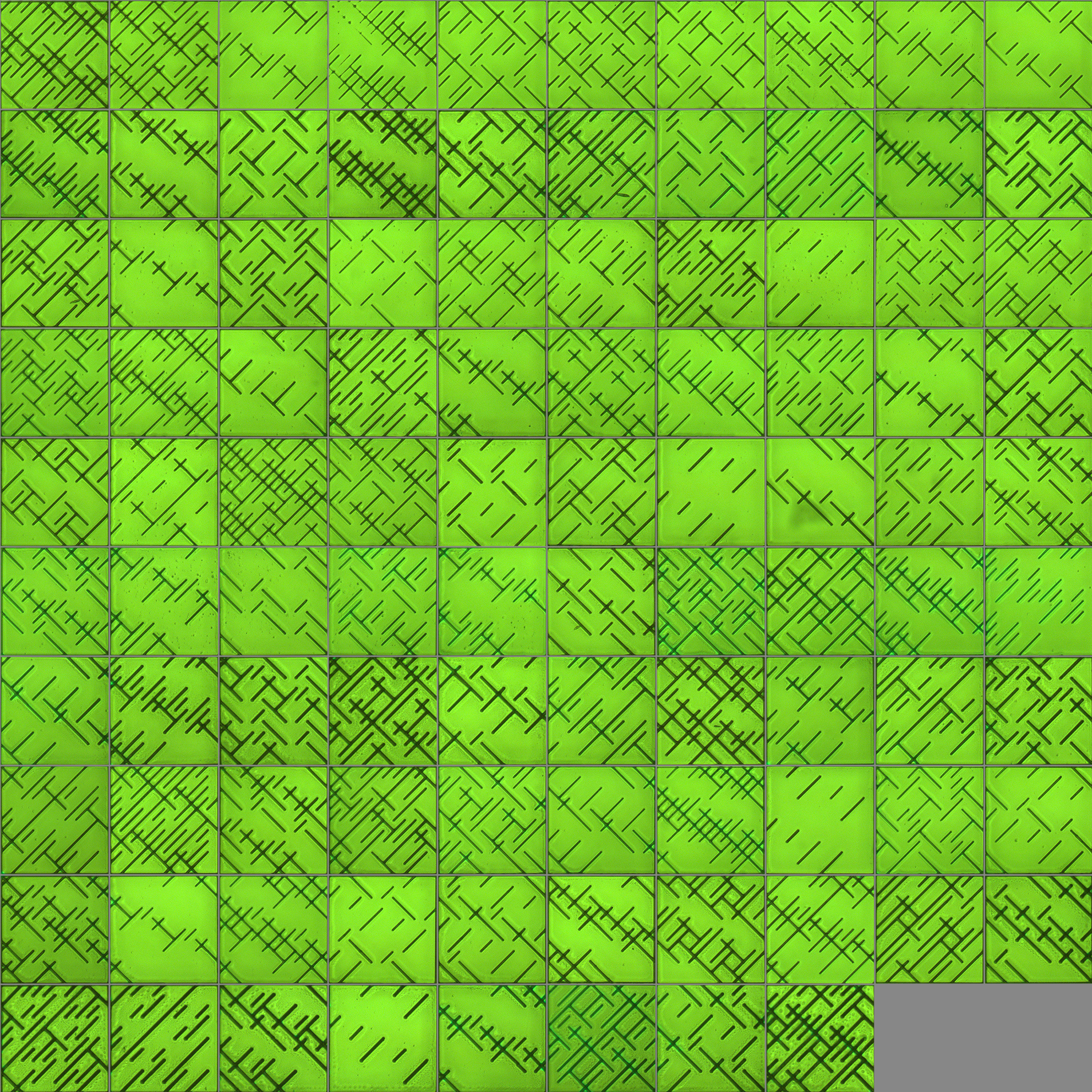


**Figure S21** The 98 completed micro-aperture array anti-counterfeit labels.
